# Evaluating the Effectiveness of Interactive Virtual Patients for Medical Education in Zambia: Randomized Controlled Trial

**DOI:** 10.2196/43699

**Published:** 2023-06-29

**Authors:** Rebecca Horst, Lea-Mara Witsch, Rayford Hazunga, Natasha Namuziya, Gardner Syakantu, Yusuf Ahmed, Omar Cherkaoui, Petros Andreadis, Florian Neuhann, Sandra Barteit

**Affiliations:** 1 Faculty of Medicine and University Hospital, Heidelberg Institute of Global Health (HIGH) Heidelberg University Heidelberg Germany; 2 Levy Mwanawasa Medical University Lusaka Zambia; 3 AMBOSS Global Health Initiative Berlin Germany; 4 SolidarMed Zambia Lusaka Zambia

**Keywords:** global health, Zambia, health care workers, medical skills, e-logbook, digital global health

## Abstract

**Background:**

Zambia is facing a severe shortage of health care workers, particularly in rural areas. Innovative educational programs and infrastructure have been established to bridge this gap; however, they encounter substantial challenges because of constraints in physical and human resources. In response to these shortcomings, strategies such as web-based and blended learning approaches have been implemented, using virtual patients (VPs) as a means to promote interactive learning at the Levy Mwanawasa Medical University (LMMU) in Zambia.

**Objective:**

This study aimed to evaluate the students’ knowledge acquisition and acceptance of 2 VP medical topics as a learning tool on a Zambian higher education e-learning platform.

**Methods:**

Using a mixed methods design, we assessed knowledge acquisition using pre- and posttests. In a randomized controlled trial setting, students were assigned (1:1) to 2 medical topics (topic 1: appendicitis and topic 2: severe acute malnutrition) and then to 4 different learning tools within their respective exposure groups: VPs, textbook content, preselected e-learning materials, and self-guided internet materials. Acceptance was evaluated using a 15-item questionnaire with a 5-point Likert scale.

**Results:**

A total of 63 third- and fourth-year Bachelor of Science clinical science students participated in the study. In the severe acute malnutrition–focused group, participants demonstrated a significant increase in knowledge within the textbook group (*P*=.01) and the VP group (*P*=.01). No substantial knowledge gain was observed in the e-learning group or the self-guided internet group. For the appendicitis-focused group, no statistically significant difference in knowledge acquisition was detected among the 4 intervention groups (*P*=.62). The acceptance of learning materials exhibited no substantial difference between the VP medical topics and other learning materials.

**Conclusions:**

In the context of LMMU, our study found that VPs were well accepted and noninferior to traditional teaching methods. VPs have the potential to serve as an engaging learning resource and can be integrated into blended learning approaches at LMMU. However, further research is required to investigate the long-term knowledge gain and the acceptance and effectiveness of VPs in medical education.

**Trial Registration:**

Pan African Clinical Trials Registry (PACTR) PACTR202211594568574; https://pactr.samrc.ac.za/TrialDisplay.aspx?TrialID=20413

## Introduction

### Background

The critical need to enhance Zambia’s health care workforce, particularly involving doctors, nurses, and other health care workers (HCWs), is driven by a substantial shortage of qualified HCWs. In this study, we define HCWs as individuals whose primary professional goal is to maintain or enhance the health of others, including those who provide patient care and diagnostic and treatment services across various clinical settings. The scarcity of HCWs is particularly acute in Zambia’s rural areas, highlighting the importance of addressing this issue to ensure equitable access to health care and improved health outcomes across the country. Addressing this HCWs deficit is essential for enhancing public health outcomes and providing equitable care across the country. Exacerbating this situation are infrastructural obstacles such as the protracted process of developing educational facilities. The convergence of these factors impedes the capacity expansion and the enhancement of health profession training quality. Consequently, addressing these constraints is vital for improving public health outcomes and fostering a robust HCW workforce. These constraints contribute to adverse public health outcomes, including compromised disease treatment efficacy, increased child mortality, and poor maternal health [1]. In 2002, Zambia introduced medical licentiate practitioners (MLPs) through an initial 3-year diploma program, followed by a 2-year medical licentiate, to address the shortage of qualified HCWs, particularly in rural areas, and to upgrade the health care delivery scope of existing clinical officers. MLPs, who have a distinct role compared with traditional medical graduates, now complete a 4-year training program, for example, at the Levy Mwanawasa Medical University (LMMU), earning a Bachelor of Science (BSc) in clinical science [2-4]. This specialized training enables MLPs to perform a limited number of emergency surgeries, including cesarean sections, and to prescribe medications [2]. Their education focuses on 4 primary disciplines that align with the country’s health priorities, particularly in rural areas: surgery, pediatrics, obstetrics and gynecology, and internal medicine [3,4]. The MLP training program comprises a balanced structure, including 2 years of theoretical instruction followed by 2 years of hands-on skills training acquired through rotational assignments at a diverse range of hospitals and health clinics across Zambia.

In low-resource learning environments, such as Zambia, e-learning and self-directed internet materials play a crucial role in overcoming the challenges posed by the scarcity of qualified HCWs, limited infrastructure, and constrained budgets. e-Learning allows for the expansion and enhancement of medical education by providing students with access to up-to-date information and resources regardless of their location. This approach is particularly beneficial for students in rural areas, where there may be a lack of qualified medical educators, limited access to learning resources, and inadequate infrastructure to support traditional face-to-face instruction [3,5].

Furthermore, self-directed internet materials encourage learners to take charge of their own education, allowing them to study at their own pace and focus on the topics most relevant to their professional development. This flexibility is crucial in low-resource settings, where e-learning can provide access to up-to-date information, reducing the reliance on on-site classes in areas facing a shortage of medical educators [3-5].

The integration of e-learning and self-directed internet materials into medical education programs in low-resource settings can help bridge the gap between theoretical knowledge and practical application, thus improving the overall quality of health care [6-8]. For example, virtual patient (VP) scenarios can provide students with a more interactive and engaging learning experience, enhancing their clinical reasoning skills and compensating for the scarcity of senior HCWs for face-to-face training [9]. By leveraging e-learning and self-directed internet materials, medical education programs in low-resource settings can better prepare students for the challenges they will face in their professional careers, ultimately leading to improved health care outcomes.

### Interactive Medical Learning Through VPs

VPs are defined by the American Association of Medical Colleges as “a specific type of computer-based program that simulates real-life clinical scenarios” [10]. VPs offer the advantage of addressing multiple cognitive levels while enhancing the learning experience through supplementary channels such as visual and auditory information. Students may develop clinical reasoning skills by using VPs, which bridge the gap between theoretical clinical knowledge and practical clinical application [3-5]. Assuming the role of a clinician, VPs enable students to practice diagnostic, treatment, and follow-up procedures. This approach may bolster student motivation as they become more aware of the importance of practice [10-12], rather than solely focusing on academic performance or examination scores [13,14]. A systematic review conducted by Kononowicz et al [11] revealed that VPs are capable of improving skills and knowledge as effectively as, or even surpassing, other prevalent educational methodologies. They observed improvements in clinical reasoning, procedural skills, and a combination of procedural and team skills in both low-income and high-income settings [11]. Bediang et al [15] assessed the impact of VP training on the clinical skills of Cameroonian health care professionals and found that such training could contribute to the advancement of users’ clinical operational skills [15].

In the Zambian context, implementing VPs as a learning resource can help mitigate challenges associated with the shortage of senior medical lecturers and infrastructure limitations. By integrating VPs into the existing e-learning platform, we can provide a more interactive and engaging learning experience, enhance clinical reasoning skills, and compensate for the scarcity of senior HCWs for face-to-face training. Furthermore, VPs can be accessed remotely, allowing students in rural areas or those with limited access to traditional educational resources to benefit from this innovative learning approach [9]. To improve medical education at LMMU within the BSc clinical sciences program, we devised and assessed 2 VP scenarios. VPs could be a highly accepted and effective tool for integration into the existing e-learning platform for MLPs at LMMU, thus expanding the range of digital learning resources available. Our study specifically focused on examining 2 primary research questions that aimed to evaluate the effectiveness and impact of these scenarios on student learning and clinical reasoning skills. By addressing these questions, we sought to provide valuable insights into the potential benefits and limitations of implementing VP scenarios in medical education, particularly within the context of low-resource settings such as Zambia. Outcomes may differ from those in high-income countries because of differences in educational systems, infrastructure, and technological access. Evaluating the potential impact of VPs in a context such as LMMU can inform targeted interventions aimed at enhancing medical education, which may ultimately contribute to improved health care outcomes within such settings.

In the context of LMMU, our study aimed to address two primary research questions: (1) How effectively do VPs contribute to knowledge acquisition in comparison with the traditional learning resources prevalent in Zambia, such as learning from textbooks, using free internet searches, and accessing preselected static resources on a medical e-learning platform? and (2) How does student acceptance of VPs compare with their acceptance of traditional textbooks, internet searches, and a medical e-learning platform as learning tools?

## Methods

### Overview

**Figure 1:**
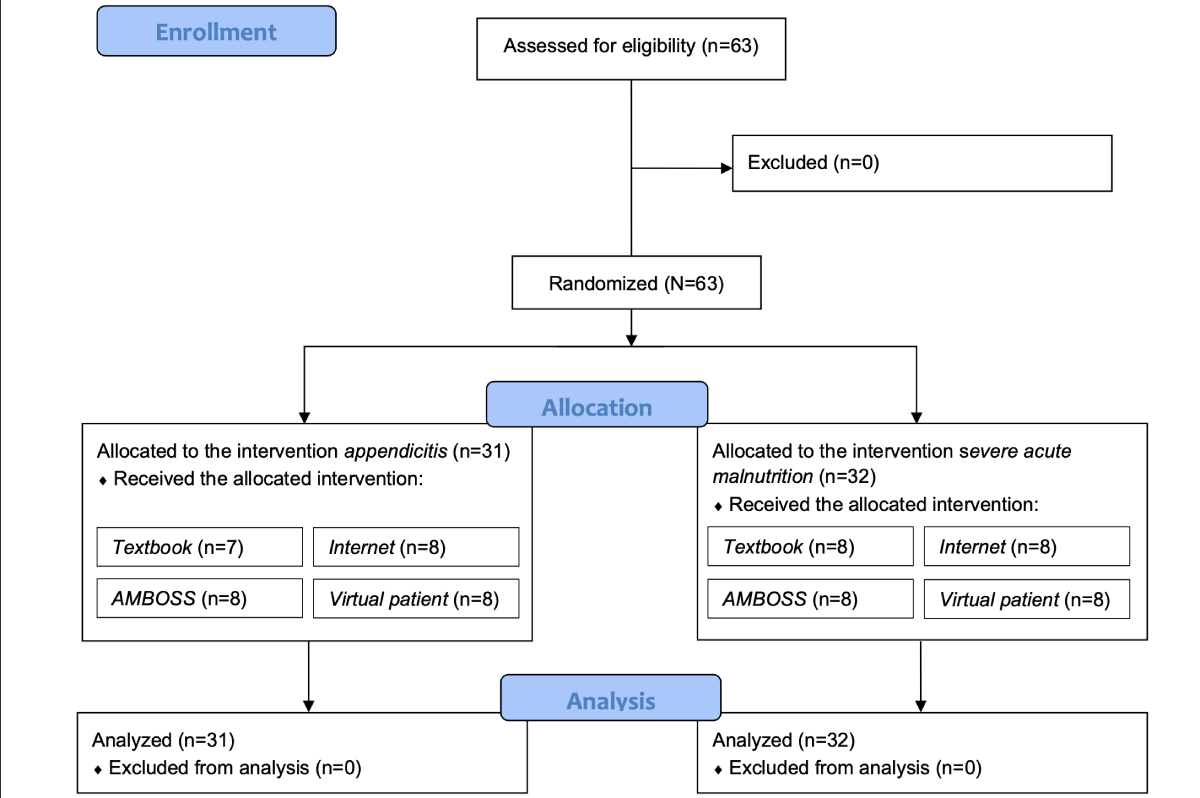
CONSORT (Consolidated Standards of Reporting Trials) flow diagram detailing the randomized controlled trial, following the stages of enrollment, allocation, and analysis.

We conducted a noninferior, randomized controlled trial with a mixed methods research design (convergent) to evaluate the effectiveness of VPs in terms of acceptance and knowledge acquisition (Figure 1). The analysis team was blinded to the study. The students, who were informed through a flyer distributed beforehand, were aware that the study aimed to investigate VPs as a learning method. The CONSORT (Consolidated Standards of Reporting Trials) checklist was used for reporting this study [16] (for the CONSORT checklist, refer to Multimedia Appendix 1). The study participants were recruited on November 29, 2021, and the study took place on December 10, 2021, at the main campus of LMMU in Lusaka, Zambia. All third- and fourth-year BSc clinical science students aged ≥18 years were eligible to participate in this study.

### Randomization, Blinding, and Implementation

The study participants were recruited through digital messaging services, email, and the local university administration. A total of 63 third- and fourth-year BSc clinical science students aged ≥18 years were invited to participate in this study. Randomization was implemented in a 2-step process. Initially, participants were assigned to 1 of the 2 study groups (appendicitis or severe acute malnutrition [SAM]) based on their study ID. Subsequently, within each study group, participants were stratified according to their academic year. We used stratified randomization to guarantee a balanced allocation of participants, considering the stratum of academic year. This procedure ensured an equitable distribution across study groups while addressing potential variations associated with participants’ academic advancement. To maintain the integrity of the study, the data analysis team remained independent of the data collection process. Only third- and fourth-year BSc clinical science students were asked to participate in the study, as they had previously been exposed to the 2 medical topics of appendicitis and SAM during their first 2 years of university training. The learning resources that the students were exposed to are presented in Textbox 1.

**Students’ learning resources.**

- Interactive virtual patient (VP) medical topics
  Severe acute malnutrition (SAM): The VP medical topic was developed using materials from the World Health Organization’s country guidelines on managing SAM in infants and children [17], web-based resources [18,19], and relevant sections from Nelson’s Textbook of Pediatrics [20] (refer to Multimedia Appendix 2 for the detailed VP medical topic).
  Appendicitis: The VP medical topic was developed using materials from the AMBOSS e-learning platform, specifically the website on appendicitis [21] (refer to Multimedia Appendix 3 for the detailed VP medical topic).
- Textbook contents aligned with the Bachelor of Science clinical science curriculum
  SAM: Nelson’s Textbook of Pediatrics, 21st edition, pages 336-352 [20].
  Appendicitis: Bailey and Love’s Short Practice of Surgery, 27th edition [22].
- e-Learning materials were preselected from the medical e-learning platform AMBOSS [23], which was made available on a complementary basis to the Levy Mwanawasa Medical University faculty and students.
- Self-guided internet materials were made accessible to study participants, allowing them to independently investigate 1 of the 2 topics (appendicitis or SAM) using their own search terms. This approach facilitates autonomous exploration and information gathering on the subject through internet resources.

Both VP medical topics were uploaded to LMMU’s Moodle e-learning platform [3] but remained inaccessible to participants until the day of the trial.

All participants, regardless of their assigned study group, completed a pretest before accessing their designated learning resource for a 30-minute period. After the intervention, a posttest identical to the pretest was administered to all the participants. Furthermore, each participant completed a questionnaire evaluating their acceptance of the respective learning resource (Multimedia Appendix 4). During the entire 4-hour study period, participants were explicitly instructed to refrain from communicating with one another.

### Data Collection

To evaluate knowledge acquisition from the 4 learning resources, we administered multiple-choice question (MCQ) tests before (pretest) and after (posttest) the intervention. The appendicitis-related MCQ test comprised 20 questions (maximum score: 1000 points), whereas the SAM-related test contained 15 questions (maximum score: 720 points; refer to Multimedia Appendix 5 for pre- and posttests). Each question was presented with 4 answer options, with 1 correct answer. All the groups received identical questions. An internal pilot study was conducted before the randomized controlled trial to ensure that participants could successfully pass the tests using any of the 4 learning resources. The pilot study involved a small sample of participants (n=6), including students (n=4) and faculty members (n=2), who were not part of the main study. The primary objective of this pilot study was to assess the clarity, comprehensibility, and effectiveness of the study materials, including the questionnaires and VPs. This preliminary testing helped to identify any potential issues, ambiguities, or biases in the questions and to evaluate the overall efficacy of the questionnaire. By addressing these concerns, we aimed to enhance the validity and reliability of the study instruments and, ultimately, the quality of the data collected in the main study.

The pilot study addressed uncertainties regarding the effectiveness and time allocation of the various study methods. This facilitated the refinement of the learning materials and ensured appropriate time durations for each method, allowing for adequate knowledge acquisition. The pilot study also confirmed that each learning resource covered course objectives, preventing participants from focusing solely on pretest questions, and enabled the assessment of pre- and posttest questions to accurately measure knowledge acquisition across learning methods. By integrating the pilot study findings into the main study design, the research team mitigated concerns about the effectiveness of various study methods and the sufficiency of the time allocated for each approach.

The VPs were developed to be consistent with the Zambian context, incorporating relevant resources and guidelines, such as the World Health Organization’s country-specific guidance on managing SAM in infants and children [17], and the curricular standards of LMMU. This method ensured that the VPs were customized to suit the requirements and expectations of BSc clinical science students as well as to address the specific demands of the local health care system.

### Acceptance Questionnaire

We assessed the acceptance of the 4 learning resources using a questionnaire adapted from the study by Davis [24] on the technology acceptance model. The technology acceptance model includes six dimensions: (1) perceived usefulness, (2) perceived ease of use, (3) attitude toward use, and (4) behavioral intention to use, (5) job relevance, and (6) perceived enjoyment. The original fifth component (actual system use) was excluded from our study’s questionnaire, as it did not pertain to our research objectives. We incorporated 2 additional dimensions—(5) and (6)—based on the study by Salloum et al [25]. The questionnaire comprised 15 items, with responses recorded on a 5-point Likert scale.

### Data Analysis

Before conducting the analyses, we examined all data for normal distribution using the Shapiro-Wilk test. *P* values <.05 were considered statistically significant. We analyzed both study groups separately. Descriptive statistics including frequency, percentage, mean, median, and SD were used to evaluate the distribution of age, sex, and prior medical knowledge within the groups. Here, prior medical knowledge refers to the participants’ preexisting knowledge specifically related to appendicitis and SAM.

The pre- and posttest outcomes were evaluated through the following analyses:

1. We used an ANOVA test followed by a paired 1-tailed *t* test with Bonferroni correction as a post hoc test to assess any variations in prior knowledge across the 4 study groups for each of the 2 study groups’ topics (appendicitis and SAM).
2. We applied the same approach described in the previous point to evaluate the differences in postintervention knowledge levels across the groups. This analysis aimed to identify whether there were any significant differences in the knowledge levels between the groups after exposure to different learning resources, which could indicate the relative effectiveness of each resource.
3. To assess within-group knowledge acquisition, we compared the pre- and posttest results for each group. We used a Wilcoxon rank test for the 3 groups using textbook contents, e-learning materials, and self-guided internet materials. For the group using VP, we used a *t* test as the data were normally distributed. This analysis helped determine the extent of knowledge gain within each group after using their assigned learning resource.

The 5-point Likert scale was converted to numerical values (1=strongly agree, 2=agree, 3=neutral, 4=disagree, and 5=strongly disagree). We assessed acceptance across the 6 dimensions using descriptive statistics. To evaluate whether there was a statistically significant difference in the acceptability of the 4 learning resources among all intervention groups, we applied the Kruskal-Wallis test. As a post hoc analysis, we conducted a Wilcoxon rank test with Bonferroni correction.

### Ethics Approval, Informed Consent, and Participation

This study was approved by the Heidelberg University Hospital Ethical Committee on August 30, 2021 (S-685/2021) and the LMMU Research Ethics Committee on November 29, 2021 (LMMU-REC 00005/21). We informed all potential and selected participants about the study’s objectives and procedures as well as their right to withdraw at any time without consequences. Before participation, each individual provided written informed consent, ensuring their voluntary involvement in the study.

## Results

### Demographics

A total of 63 students consented to participate and were included in the study. The mean age of the participants was 39.56 (SD 6.05) years, with age ranging from 22 to 46 years. The sample consisted of 39 male students and 23 female students, with 1 participant identifying as “diverse.” Regarding the participants’ academic progression, 32 were in their third year of study, whereas 31 were in their fourth year (refer to Table 1 for a detailed breakdown).

**Table 1:** Overview of the study groups’ composition and demographic characteristics (N=63).

Intervention group		Participants, n	Age (years), mean (SD)		Sex, n (%)						Study year, n (%)		
					Female	Male		Diverse		Third			Fourth
**Study group 1—medical topic: severe acute malnutrition (n=32)**													
	Virtual patient	8	29.38 (1.77)	4 (50)		4 (50)	0 (0)		4 (50)			4 (50)	
	Textbook	8	27 (5.04)	3 (38)		5 (62)	0 (0)		4 (50)			4 (50)	
	Preselected e-learning materials	8	30,38 (6.82)	4 (50)		3 (38)	1 (12)		4 (50)			4 (50)	
	Self-guided internet materials	8	32.75 (9.66)	0 (0)		8 (100)	0 (0)		4 (50)			4 (50)	
**Study group 2—medical topic: appendicitis (n=31)**													
	Virtual patient	8	29.39 (4.57)	5 (62)		3 (57)	0 (0)		4 (50)			4 (50)	
	Textbook	7	29.17 (3.87)	2 (29)		5 (71)	0 (0)		4 (57)			3 (43)	
	Preselected e-learning materials	8	33.75 (4.74)	1 (13)		7 (87)	0 (0)		4 (50)			4 (50)	
	Self-guided internet materials	8	32.28 (7.51)	4 (50)		4 (50)	0 (0)		4 (50)			4 (50)	

### MCQ Pre- and Posttests

#### Pre- and Posttests for SAM

The pretest revealed a significant difference in knowledge between the *VP group* (mean score 480, SD 76.97) and the *textbook group* (mean score 456, SD 67.88) participants (*P*=.01) as well as a difference between the *VP group* and *self-guided* *internet group* (*P*=.05; refer to Table 2 for detailed results). Participants in the *VP group* achieved the highest pretest scores, with a mean of 68% (43/63; mean score 480, SD 76.97) of the questions correctly answered, followed closely by the *e-learning group,* who correctly answered 63% (40/63) of the questions (mean score 456, SD 67.88). Students in the *self-guided* *internet group* scored 50% (mean score 360, SD 105.79), whereas students in the *textbook group* scored 48% (mean score 342, SD 82.89). Although differences in scores between the groups persisted in the posttest compared with the pretest, the overall knowledge gap between the groups narrowed: The *VP group* scored 79% (mean score 570, SD 82.89), the *e-learning group* scored 68% (mean score 468, SD 132.7), the *self-guided* *internet group* scored 59% (mean score 426, SD 165.16), and the *textbook group* scored 59% (mean score 426, SD 82.89; refer to Table 2 for details).

No significant increase in knowledge was observed in the *e-learning group* (mean score 468, SD 132.7) and the *self-guided* *internet group* (mean score 426, SD 165.16). However, significant knowledge growth was identified in both the *textbook group* (*P*=.01) and the *VP group* (*P*=.01; refer to Multimedia Appendix 6 for details).

**Table 2:** Overview of the 4 exposure intervention groups that were exposed to the medical topic of severe acute malnutrition as well as their relative test scores (pre- and posttest) and average knowledge increase.

Intervention group (severe acute malnutrition)	Pretest score, mean (SD)	Posttest score, mean (SD)	Knowledge gain score, mean (SD)
Virtual patient	480 (76.97)	570 (82.89)	90 (90.48)
Textbook contents	342 (82.89)	426 (82.89)	84 (80.11)
Preselected e-learning materials	456 (67.88)	468 (132.7)	12 (114.02)
Self-guided internet materials	360 (105.79)	426 (165.16)	66 (122.88)

#### Pre- and Posttests for Appendicitis

The pretest for the medical topic of appendicitis did not reveal a significant difference in knowledge acquisition between the 4 intervention groups (*P*=.62; refer to Table 3 for detailed results). Participants in the *e-learning group* achieved the highest pretest score, with a mean score 75% (mean score 750, SD 46.29), closely followed by participants in the *self-guided* *internet group* with 74% (mean score 73.5, SD 112.6), the *textbook group* with 74% (mean score 735, SD 146.3), and the *VP group* with 72% (mean score 718, SD 106.7). In the posttest, a difference was observed between the intervention groups, although this difference was not substantial (Table 3). On the basis of the individual learning curves, moderate knowledge acquisition was observed in all 4 groups, although these changes were not substantial (refer to Multimedia Appendix 7 for details).

**Table 3:** Overview of the 4 exposure intervention groups that were exposed to the medical topic of appendicitis as well as their relative test scores (pre- and posttest) and average knowledge increase.

Intervention group (appendicitis)	Pretest score, mean (SD)	Posttest score, mean (SD)	Knowledge acquisition, mean (SD)
Virtual patient	718 (106.7)	800 (128.17)	81.25 (106.7)
Textbook	735 (146.3)	821.43 (128.64)	85.71 (85.22)
Preselected e-learning materials	750 (46.29)	887.5 (74.4)	137.5 (87.63)
Self-guided internet materials	735 (112.6)	850 (75.59)	112.5 (99.1)

### Acceptance Questionnaire

We observed that the acceptance of learning resources varied depending on the medical topic. For the topic of SAM, the response to the statement “If given the opportunity, I would favor this learning resource over others” showed a significant difference between the *e-learning group* (mean 2.5, SD 0.33; *P*=.01) and the *self-guided internet group* (mean 1.25, SD 0.46). In contrast, the *VP group* had a favorable score (mean 1.62, SD 0.72). For the topic of appendicitis, the mean response to the same statement in the *e-learning group* was 1.38 (SD 0.52), which is significantly lower than the mean response in the *VP group* (mean 3.62, SD 1.41; *P*=.02).

Regarding the statement “I think this learning resource is a good instrument to acquire knowledge,” a difference between the *e-learning group* (mean 1.5, SD 0.53) and the *self-guided internet group* (mean 3.12, SD 1.13) with *P*=.02 was observed for the topic of appendicitis. This finding indicated that the *e-learning group* received more positive feedback than the *self-guided internet group* (Table 4).

One misinterpreted question in the study was removed because of its outlier status (question item: “Interacting with the learning mode required considerable effort.”).

**Table 4:** The acceptance questionnaire results for both study groups by intervention groups showing mean and SD.

Study arm and questions items (acceptance questionnaire)		Intervention groups, mean (SD)			
		Virtual patient group	Textbook group	Preselected e-learning materials	Self-guided internet materials
**Study arm 1: severe acute malnutrition**					
	“I think this learning resource is a good instrument to acquire knowledge.”	1.75 (0.89)	1.88 (1.13)	2.00 (0.76)	1.62 (0.52)
	“If given the opportunity, I would favor this learning resource over others.”	1.62 (0.74)	2.62 (1.19)	2.5 (0.53)	1.25 (0.46)
**Study arm 2: appendicitis**					
	“I think this learning resource is a good instrument to acquire knowledge.”	2.12 (0.64)	1.4 (0.89)	1.5 (0.53)	3.12 (1.13)
	“If given the opportunity I would favor this learning resource over others.”	3.62 (1.41)	2.00 (0.82)	1.38 (0.52)	2.75 (1.04)

## Discussion

### Principal Findings

In our study, we developed and assessed 2 interactive VP medical topics focusing on SAM and appendicitis, aiming to evaluate their effectiveness and acceptance compared with other prevalent learning resources at LMMU. The efficacy of transferring knowledge to students, and the precise impact of certain VP features, had previously been ambiguous. Our study aimed to address these aspects.

The primary aim of this study was to evaluate the acceptance and knowledge acquisition of BSc clinical sciences students at LMMU when using VPs as a learning resource in comparison with textbooks, preselected e-learning materials, and self-guided internet materials. A key finding of this study was that all 4 learning resources demonstrated their effectiveness in promoting knowledge gain within the study setting. Furthermore, VPs were well received by the students and proved to be noninferior compared with the other 3 learning methods.

### Comparison With Prior Work

Knowledge acquisition significantly increased in *the VP and textbook groups* but not in the *e-learning* or *self-guided internet groups*. The differences in knowledge acquisition between these groups can be attributed to various factors. Each learning resource provides different levels of structure and guidance, with some students preferring visual or interactive content (eg, VPs) and others opting for text-based resources (eg, textbooks). These individual preferences may influence the effectiveness of each learning method, thus impacting knowledge acquisition across the groups. Motivation and engagement may also play a role, as VPs and textbooks potentially offer a more structured and engaging learning experience, which could lead to increased motivation and improved information retention. In contrast, general e-learning and self-guided internet materials may require a higher degree of self-discipline and motivation to effectively navigate and absorb the content. Another aspect to consider is the familiarity with learning resources. Students might be more familiar with traditional learning resources, such as textbooks, compared with newer methods, such as VPs or e-learning platforms. This familiarity could influence the ease with which students can use and learn from these resources, thus affecting their knowledge acquisition. Finally, access to specific, targeted learning materials is essential. The *VP* and *textbook groups* had access to well-organized, systematic learning materials, making it easier for students to focus on relevant content and efficiently grasp key concepts. In contrast, participants in the e-learning and self-guided internet groups had to navigate and search for pertinent information independently. This process could be time consuming and challenging, as students might encounter a vast amount of information of varying complexity that is not always directly related to course objectives. Consequently, these students may have faced difficulties in identifying and assimilating the critical knowledge required for the subject matter, leading to a smaller increase in knowledge acquisition compared with their counterparts in the *VP* and *textbook groups*. However, this observation was not reflected in the acceptance questionnaire. In the second trial group (appendicitis), the pre- and posttest results revealed no significant differences in knowledge acquisition among participants in the intervention groups, although all 4 groups demonstrated an increase in knowledge. The acceptance questionnaires indicated similar responses for all 4 learning resources across 6 technology acceptance dimensions (perceived usefulness, perceived ease of use, attitude toward using, behavioral intention to use, job relevance, and perceived enjoyment) but showed mixed results when comparing the 2 medical subjects of SAM and appendicitis.

Participants exposed to the SAM VP intervention displayed a higher preference for this learning resource, which was not observed in the group exposed to the appendicitis VP. This difference could be attributed to the SAM VP’s integration of more images and a visually appealing design, making it more engaging for students. The varying success of VPs for the 2 subjects may be attributed to differences in content and design. The appendicitis VP might have lacked the engaging elements found in the SAM VP, leading to a lower preference among participants. In addition, the participants’ higher familiarity with or the lower complexity of 1 topic could have contributed to the observed differences in the success of the respective VPs. However, it is important to consider that the design of the 2 VP medical topics may have acted as a confounding factor, potentially influencing the outcomes. As such, we cannot definitively attribute the observed differences solely to the subject matter or the VP medical topic design. Further research is needed to identify the specific factors that contribute to the success of VPs in medical education.

In the SAM group, the *preselected e-learning materials* (AMBOSS platform) received the lowest mean rating among all intervention groups. Conversely, the appendicitis group demonstrated a positive response to the AMBOSS platform but displayed indifference toward self-guided web-based learning materials. The disparity in the acceptance of the AMBOSS platform as a learning resource between the 2 study groups might be attributed to the platform’s content, which primarily targets the global north.

The content of the AMBOSS platform may not be adequately tailored to the specific learning needs and objectives of the SAM group, possibly because of differences in guidelines, treatment protocols, or context-specific challenges in managing SAM between the United States and Zambia. In addition, the AMBOSS platform may use terminology, examples, or scenarios predominantly familiar to US-based learners, which could pose comprehension difficulties for Zambian students when addressing the specific topic of SAM. SAM is a pressing issue in Zambia, with treatment priorities and modalities that may differ from those in the United States. In contrast, appendicitis holds similar importance in both countries.

### Pre- and Posttests

Upon comparing the pre- and posttest results of all study participants, the most substantial improvement was observed among fourth-year students. This finding aligns with the study by Kiesewetter et al [26], who reported that students with less prior knowledge experienced a greater cognitive load compared with those with more prior knowledge. A potential advantage of using VPs is their accessibility, as textbooks can be expensive, sometimes scarce, and may contain outdated content when published. Incorporating VPs into the curriculum can help overcome these limitations and provide students with up-to-date and readily available learning resources. Overall, our pre- and posttest findings indicate that VPs are as effective in promoting learning as other widely used learning resources. Previous research has indicated that the use of VPs leads to significant increases in knowledge, enhanced understanding, and improved problem-solving skills when compared with lecture-based small seminar groups [3,27,28]. These studies also evaluated long-term knowledge retention, revealing no discernible differences between the 2 groups over a 4- to 6-week period.

### Acceptance Questionnaire

The observed disparity in the acceptance of VPs between the 2 study groups, SAM and appendicitis, could potentially be attributed to the differences in the design of the 2 VP medical topics. Peddle et al [29] conducted a study involving student interviews to better understand the acceptance of VPs and discovered that incorporating images improved student comprehension and facilitated knowledge retention. The study emphasized the benefits of using short videos to promote knowledge acquisition.

In general, responses to the acceptance questionnaire were predominantly positive. Participants frequently selected responses that ranged from positive to neutral, whereas negative responses were rare. This pattern was also observed in other studies [30]. The study by Krumpal [31] described this phenomenon as individuals considering risks and losses when determining a response, as they seek social acceptability.

To address this potential bias, we communicated with the participants before the study, emphasizing that their responses to the questionnaires would be handled anonymously and would not affect their academic performance.

Future studies should carefully control for potential confounding factors, such as differences in design, when examining the effectiveness of VPs across different medical subjects. This would allow for a more accurate assessment of the impact of subject matter and case design on learning outcomes.

### Strengths and Limitations

Our study, which encompassed a majority of students and investigated 4 distinct learning methods, provides valuable insights into how these approaches might improve student learning. However, several limitations of this study should be acknowledged.

First, the generalizability of our findings is limited because of the study population, which exclusively consisted of third- and fourth-year BSc clinical science students. Consequently, our results may not be directly applicable to other contexts, such as different disciplines.

Second, the content of the AMBOSS platform may not have been adequately tailored to the specific learning needs and objectives of SAM in Zambia. Disparities in the content and design of VP between the 2 case scenarios may also result in differences in the study outcomes. Future studies should consider developing a more robust method for comparing VP cases, such as using a larger sample of case scenarios or ensuring that the cases are matched in terms of difficulty and complexity.

Third, owing to a 1-hour delay at the study’s onset caused by technical issues, some participants might have experienced time pressure during the later stages (posttest and acceptance questionnaire), potentially introducing bias. To mitigate this concern, we requested all participants to wait until the last participant completed the study. However, the delay could have resulted in increased fatigue among the participants, affecting their concentration, motivation, and overall performance during the learning sessions and the pre- and posttests. This factor could have potentially impacted knowledge acquisition, leading to lower scores across all intervention groups. Moreover, the delay may have induced stress or frustration that could have influenced their approach to the learning sessions and the pre- and posttests, resulting in less accurate or reliable data and affecting the overall interpretation of the study results.

Fourth, the study did not examine long-term knowledge retention or evaluate traits such as clinical reasoning using MCQs. Therefore, additional research is warranted to investigate long-term knowledge acquisition, as this study concentrated solely on immediate knowledge gain.

Fifth, in our study, we acknowledge the possibility that participants may have been hesitant to provide negative feedback because of concerns regarding anonymity and potential implications for their academic performance. To address this concern, we implemented several measures to ensure anonymity of the data collected. These measures included (1) emphasizing the confidentiality of the study in the information provided to the participants, both verbally and in written form; (2) assigning unique participant identification numbers, which were not linked to personal information, to protect the identity of the participants during data collection and analysis; (3) ensuring that the questionnaires were completed individually and without peer or instructor influence; and (4) storing the collected data securely and restricting access to only the researchers directly involved in the study.

By implementing these measures, we aimed to minimize any potential bias arising from the participants’ reluctance to provide negative feedback. Nevertheless, it is important to recognize that a certain degree of social desirability bias may still be present, which is common in self-reporting studies. Future research could explore alternative methods of data collection or use more indirect questioning techniques to further reduce the impact of such biases on the study results.

### Future Directions

At present, LMMU is in the process of revising its e-learning strategy, with the aim of fully integrating e-learning into the curriculum in the future. In light of the findings from our study, it is important to consider potential methodological concerns, such as the content of the AMBOSS platform not being adequately tailored to the specific learning needs and objectives of SAM for Zambia, as well as disparities in the content and design of VP between the 2 case scenarios that may result in differences in study outcomes. Despite these concerns, the updated e-learning strategy and our study results may support the potential inclusion of VPs within the curriculum as a means to enhance medical education at LMMU. Future research should address these methodological concerns to ensure that the implementation of VP scenarios is tailored to the specific needs and contexts of medical education in Zambia, ultimately leading to more robust and reliable outcomes.

### Conclusions

The primary aim of this study was to assess the acceptability and effectiveness of VPs for knowledge acquisition in the BSc clinical science program at LMMU in Zambia, comparing their performance with 3 other prevalent learning resources: textbook content, preselected e-learning materials, and self-guided internet materials. In the context of a low-resource setting, our findings demonstrate that although VPs are well accepted, their effectiveness in terms of knowledge acquisition may vary depending on the specific case scenario and content design.

These results underscore the importance of adapting VP designs to Zambian needs and addressing the limitations observed in the appendicitis case before making broad statements regarding their comparability with other learning methods. When appropriately tailored to local contexts, VPs can function as an engaging and interactive learning strategy to enhance web-based or blended learning programs in settings with a high student-to-faculty ratio and limited teaching resources.

Nonetheless, further research on the acceptability and effectiveness of VPs is warranted, as the incorporation of additional VP medical topics into the blended learning program at LMMU for BSc clinical science students is planned. Expanding the evidence base will ensure that VPs continue to contribute positively to medical education in low-resource settings, support the ongoing development and refinement of these learning resources, and address potential disparities in content and design that may impact their effectiveness.

## Appendix

Multimedia Appendix 1 CONSORT eHEALTH checklist (V 1.6.1).

Multimedia Appendix 2 Virtual patient medical topic: severe acute malnutrition.

Multimedia Appendix 3 Virtual patient medical topic: appendicitis.

Multimedia Appendix 4 Acceptance questionnaire.

Multimedia Appendix 5 Knowledge pre- and posttests.

Multimedia Appendix 6 Individual learning trajectories of study participants in the context of severe acute malnutrition.

Multimedia Appendix 7 Individual learning trajectories of study participants in the context of appendicitis.

## Abbreviations

BSc: Bachelor of Science
CONSORT: Consolidated Standards of Reporting Trials
HCW: health care worker
LMMU: Levy Mwanawasa Medical University
MCQ: multiple-choice question
MLP: medical licentiate practitioner
SAM: severe acute malnutrition
VP: virtual patient

## Data Availability

The data sets generated and analyzed during this study are not publicly available because of data protection regulations but are available from the corresponding author upon reasonable request.
