# Supplementary material for: Evaluating the Effectiveness of Interactive Virtual Patients for Medical Education in Zambia: Randomized Controlled Trial
Source: JMIR Med Educ. 2023 Jun 29;9:e43699. doi: 10.2196/43699 (PMC10501501; doi:10.2196/43699)
Supplement: Multimedia Appendix 1 [file mededu_v9i1e43699_app1.pdf]

CONSORT-EHEALTH Checklist V1.6.2 Report	Manuscript Number	43699

(based on CONSORT-EHEALTH V1.6), available at [http://tinyurl.com/consort-ehealth-v1-6].

Date completed
6/19/2023 13:30:13
by
Lea-Mara Witsch

Evaluating the Effectiveness of Interactive Virtual Patients for
Medical Education in Zambia: Randomized Controlled Trial

TITLE

1a-i) Identify the mode of delivery in the title

"Evaluating the Effectiveness of Interactive Virtual Patients for
Medical Education in Zambia"

1a-ii) Non-web-based components or important co-interventions in title

The main focus was the evaluation of virtual patient cases and therefore no non-web-based components are mentioned in the title

1a-iii) Primary condition or target group in the title

The title mentions the target group "medical education", which is a very general description of a large target group not yet specified more clearly.

ABSTRACT

1b-i) Key features/functionalities/components of the intervention and comparator in the METHODS section of the ABSTRACT

"In response to these shortcomings, strategies such as web-based and blended learning
approaches have been implemented, using virtual patients (VPs) as a means to promote interactive learning at the Levy Mwanawasa
Medical University (LMMU) in Zambia."

1b-ii) Level of human involvement in the METHODS section of the ABSTRACT

"In a randomized controlled trial setting, students were assigned (1:1) to 2 medical topics (topic 1: appendicitis and topic 2: severe acute malnutrition) and then to 4 different learning tools within their respective exposure groups"

1b-iii) Open vs. closed, web-based (self-assessment) vs. face-to-face assessments in the METHODS section of the ABSTRACT

The method section of the abstract does not clearly describe the form of recruitement, since the participants were all recruited from a small group of students studying at one university. It does describe however the different forms of exposure, including web-based and non-web-based learning materials: "In a randomized controlled trial setting, students were assigned (1:1) to 2 medical topics (topic 1: appendicitis and topic 2: severe acute malnutrition) and then to 4 different learning tools within their respective exposure groups: VPs, textbook content, preselected e-learning materials, and self-guided internet materials."

1b-iv) RESULTS section in abstract must contain use data

"In the severe acute malnutrition–focused group, participants demonstrated a significant increase in knowledge within the textbook group (P=.01) and the VP group (P=.01). No substantial knowledge gain was observed in the e-learning group or the self-guided internet
group. For the appendicitis-focused group, no statistically significant difference in knowledge acquisition was detected among the 4 intervention groups (P=.62). The acceptance of learning materials exhibited no substantial difference between the VP
medical topics and other learning materials"

1b-v) CONCLUSIONS/DISCUSSION in abstract for negative trials

INTRODUCTION

2a-i) Problem and the type of system/solution

"Our study specifically focused on examining 2 primary research questions that aimed to evaluate the effectiveness and impact of these scenarios on student learning and clinical reasoning skills. By addressing these questions, we sought to provide valuable insights into the potential benefits and limitations of implementing VP scenarios in medical education, particularly within the context of low-resource settings such as Zambia. Outcomes may differ from those in high-income countries because of differences in educational systems, infrastructure, and technological access. Evaluating the potential impact of VPs in a context such as LMMU can inform targeted interventions aimed at enhancing medical education, which may ultimately contribute to improved health care outcomes within such settings."

2a-ii) Scientific background, rationale: What is known about the (type of) system

"In low-resource learning environments, such as Zambia, e-learning and self-directed internet materials play a crucial role in overcoming the challenges posed by the scarcity of qualified HCWs, limited infrastructure, and constrained budgets. e-Learning allows for the expansion and enhancement of medical education by providing students with access to up-to-date information and resources regardless of their location. This approach is particularly beneficial for students in rural areas, where there may be a lack of qualified medical educators, limited access to learning resources, and inadequate infrastructure to support traditional face-to-face instruction"

Does your paper address CONSORT subitem 2b?

"In the context of LMMU, our study aimed to address two primary research questions: (1) How effectively do VPs contribute to knowledge acquisition in comparison with the traditional learning resources prevalent in Zambia, such as learning from textbooks, using free internet searches, and accessing preselected static resources on a medical e-learning platform? and (2) How does student acceptance of VPs compare with their acceptance of traditional textbooks, internet searches, and a medical e-learning platform as learning tools?"

METHODS

3a) CONSORT: Description of trial design (such as parallel, factorial) including allocation ratio

"A total of 63 third- and fourth-year BSc clinical science students aged ≥18 years were invited to participate in this study. Randomization was implemented in a 2-step process. Initially, participants were assigned to 1 of the 2 study groups (appendicitis or severe acute malnutrition [SAM]) based on their study ID. Subsequently, within each study group, participants were stratified according to their academic year"

"We conducted a noninferior, randomized controlled trial with a mixed methods research design (convergent) to evaluate the effectiveness of VPs in terms of acceptance and knowledge acquisition (Figure 1). The analysis team was blinded to the study."

3b) CONSORT: Important changes to methods after trial commencement (such as eligibility criteria), with reasons

"Third, owing to a 1-hour delay at the study's onset caused by technical issues, some participants might have experienced time pressure during the later stages (posttest and acceptance questionnaire), potentially introducing bias"

3b-i) Bug fixes, Downtimes, Content Changes

"Third, owing to a 1-hour delay at the study's onset caused by<br>technical issues, some participants might have experienced time<br>pressure during the later stages (posttest and acceptance<br>questionnaire), potentially introducing bias"		
**4a) CONSORT: Eligibility criteria for participants**		
"All third- and fourth-year BSc clinical science students<br>aged ≥18 years were eligible to participate in this study"		
**4a-i) Computer / Internet literacy**		
**4a-ii) Open vs. closed, web-based vs. face-to-face assessments:**		
"The study participants were<br>recruited on November 29, 2021, and the study took place on<br>December 10, 2021, at the main campus of LMMU in Lusaka,<br>Zambia. All third- and fourth-year BSc clinical science students<br>aged ≥18 years were eligible to participate in this study"<br><br>"The analysis team was blinded to the<br>study. The students, who were informed through a flyer<br>distributed beforehand, were aware that the study aimed to<br>investigate VPs as a learning method"		
**4a-iii) Information giving during recruitment**		
"We informed all potential and selected<br>participants about the study's objectives and procedures as well<br>as their right to withdraw at any time without consequences.<br>Before participation, each individual provided written informed<br>consent, ensuring their voluntary involvement in the study"		
**4b) CONSORT: Settings and locations where the data were collected**		
"The study participants were<br>recruited on November 29, 2021, and the study took place on<br>December 10, 2021, at the main campus of LMMU in Lusaka,<br>Zambia. All third- and fourth-year BSc clinical science students<br>aged ≥18 years were eligible to participate in this study"		
**4b-i) Report if outcomes were (self-)assessed through online questionnaires**		
"All participants, regardless of their assigned study group,<br>completed a pretest before accessing their designated learning<br>resource for a 30-minute period. After the intervention, a posttest<br>identical to the pretest was administered to all the participants.<br>Furthermore, each participant completed a questionnaire<br>evaluating their acceptance of the respective learning resource"		
**4b-ii) Report how institutional affiliations are displayed**		
"This study was approved by the Heidelberg University Hospital<br>Ethical Committee on August 30, 2021 (S-685/2021) and the<br>LMMU Research Ethics Committee on November 29, 2021<br>(LMMU-REC 00005/21). We informed all potential and selected<br>participants about the study's objectives and procedures as well<br>as their right to withdraw at any time without consequences.<br>Before participation, each individual provided written informed<br>consent, ensuring their voluntary involvement in the study"		
**5) CONSORT: Describe the interventions for each group with sufficient details to allow replication, including how and when they were actually administered**		
**5-i) Mention names, credential, affiliations of the developers, sponsors, and owners**		
"This work was supported by the Else Kröner-Fresenius-Stiftung (2019_HA25). For the publication fee, the authors acknowledge financial support from the Deutsche Forschungsgemeinschaft within the funding program "Open Access Publikationskosten" as<br>well as from the Heidelberg University. The authors would like to thank Engagement Global GmbH for their support within the framework of the ASA program"		
**5-ii) Describe the history/development process**		
"An internal pilot study was conducted before the randomized controlled trial to<br>ensure that participants could successfully pass the tests using any of the 4 learning resources"		
**5-iii) Revisions and updating**		
"The pilot study addressed uncertainties regarding the<br>effectiveness and time allocation of the various study methods.<br>This facilitated the refinement of the learning materials and<br>ensured appropriate time durations for each method, allowing<br>for adequate knowledge acquisition. The pilot study also<br>confirmed that each learning resource covered course objectives,<br>preventing participants from focusing solely on pretest<br>questions, and enabled the assessment of pre- and posttest<br>questions to accurately measure knowledge acquisition across<br>learning methods"		
**5-iv) Quality assurance methods**		
"To evaluate whether<br>there was a statistically significant difference in the acceptability<br>of the 4 learning resources among all intervention groups, we<br>applied the Kruskal-Wallis test. As a post hoc analysis, we<br>conducted a Wilcoxon rank test with Bonferroni correction"		
**5-v) Ensure replicability by publishing the source code, and/or providing screenshots/screen-capture video, and/or providing flowcharts of the algorithms used**		
The Appendix of our study does include all questionaires and interventional sources used during the trial.		
**5-vi) Digital preservation**		
All interventional resources and questionaires used durin the study are saved in the appendix of our article.		
**5-vii) Access**		
"Both VP medical topics were uploaded to LMMU's Moodle e-learning platform [3] but remained inaccessible to participants until the day of the trial"		
**5-viii) Mode of delivery, features/functionalities/components of the intervention and comparator, and the theoretical framework**		
"Interactive virtual patient (VP) medical topics<br>• Severe acute malnutrition (SAM): The VP medical topic was developed using materials from the World Health Organization's country<br>guidelines on managing SAM in infants and children [17], web-based resources [18,19], and relevant sections from Nelson's Textbook of<br>Pediatrics [20] (refer to Multimedia Appendix 2 for the detailed VP medical topic).<br>• Appendicitis: The VP medical topic was developed using materials from the AMBOSS e-learning platform, specifically the website on<br>appendicitis [21] (refer to Multimedia Appendix 3 for the detailed VP medical topic).<br>• Textbook contents aligned with the Bachelor of Science clinical science curriculum<br>• SAM: Nelson's Textbook of Pediatrics, 21st edition, pages 336-352 [20].<br>• Appendicitis: Bailey and Love's Short Practice of Surgery, 27th edition [22].<br>• e-Learning materials were preselected from the medical e-learning platform AMBOSS [23], which was made available on a complementary basis<br>to the Levy Mwanawasa Medical University faculty and students.<br>• Self-guided internet materials were made accessible to study participants, allowing them to independently investigate 1 of the 2 topics (appendicitis<br>or SAM) using their own search terms. This approach facilitates autonomous exploration and information gathering on the subject through internet<br>resources."		
**5-ix) Describe use parameters**		
In this study participants were only exposed to the different interventions during the time of the trial (4 hours).		
**5-x) Clarify the level of human involvement**		
**5-xi) Report any prompts/reminders used**		
"The study participants were recruited through digital messaging services, email, and the local university administration"		
**5-xii) Describe any co-interventions (incl. training/support)**		

There were no co-interventions done during, before or after our trial.		
**6a) CONSORT: Completely defined pre-specified primary and secondary outcome measures, including how and when they were assessed**		
"We used an ANOVA test followed by a paired 1-tailed t<br>test with Bonferroni correction as a post hoc test to assess<br>any variations in prior knowledge across the 4 study groups<br>for each of the 2 study groups' topics (appendicitis and<br>SAM).<br>2. We applied the same approach described in the previous<br>point to evaluate the differences in postintervention<br>knowledge levels across the groups. This analysis aimed<br>to identify whether there were any significant differences<br>in the knowledge levels between the groups after exposure<br>to different learning resources, which could indicate the<br>relative effectiveness of each resource.<br>3. To assess within-group knowledge acquisition, we<br>compared the pre- and posttest results for each group. We<br>used a Wilcoxon rank test for the 3 groups using textbook<br>contents, e-learning materials, and self-guided internet<br>materials. For the group using VP, we used a t test as the<br>data were normally distributed. This analysis helped<br>determine the extent of knowledge gain within each group<br>after using their assigned learning resource."		
**6a-i) Online questionnaires: describe if they were validated for online use and apply CHERRIES items to describe how the questionnaires were designed/deployed**		
**6a-ii) Describe whether and how "use" (including intensity of use/dosage) was defined/measured/monitored**		
**6a-iii) Describe whether, how, and when qualitative feedback from participants was obtained**		
"We assessed the acceptance of the 4 learning resources using a<br>questionnaire adapted from the study by Davis [24] on the<br>technology acceptance model"		
**6b) CONSORT: Any changes to trial outcomes after the trial commenced, with reasons**		
"The study participants were<br>recruited on November 29, 2021, and the study took place on<br>December 10, 2021, at the main campus of LMMU in Lusaka,<br>Zambia. All third- and fourth-year BSc clinical science students<br>aged ≥18 years were eligible to participate in this study"		
**7a) CONSORT: How sample size was determined**		
**7a-i) Describe whether and how expected attrition was taken into account when calculating the sample size**		
**7b) CONSORT: When applicable, explanation of any interim analyses and stopping guidelines**		
"We used an ANOVA test followed by a paired 1-tailed t<br>test with Bonferroni correction as a post hoc test to assess<br>any variations in prior knowledge across the 4 study groups<br>for each of the 2 study groups' topics (appendicitis and<br>SAM).<br>2. We applied the same approach described in the previous<br>point to evaluate the differences in postintervention<br>knowledge levels across the groups. This analysis aimed<br>to identify whether there were any significant differences<br>in the knowledge levels between the groups after exposure<br>to different learning resources, which could indicate the<br>relative effectiveness of each resource.<br>3. To assess within-group knowledge acquisition, we<br>compared the pre- and posttest results for each group. We<br>used a Wilcoxon rank test for the 3 groups using textbook<br>contents, e-learning materials, and self-guided internet<br>materials. For the group using VP, we used a t test as the<br>data were normally distributed. This analysis helped<br>determine the extent of knowledge gain within each group<br>after using their assigned learning resource."		
**8a) CONSORT: Method used to generate the random allocation sequence**		
"Initially, participants were assigned to 1 of the 2 study groups<br>(appendicitis or severe acute malnutrition [SAM]) based on<br>their study ID. Subsequently, within each study group,<br>participants were stratified according to their academic year.<br>We used stratified randomization to guarantee a balanced<br>allocation of participants, considering the stratum of academic<br>year. This procedure ensured an equitable distribution across<br>study groups while addressing potential variations associated<br>with participants' academic advancement."		
**8b) CONSORT: Type of randomisation; details of any restriction (such as blocking and block size)**		
"Only third- and fourth-year BSc clinical science students were asked to<br>participate in the study, as they had previously been exposed<br>to the 2 medical topics of appendicitis and SAM during their<br>first 2 years of university training"<br><br>"A total of 63 third- and fourth-year BSc clinical science students aged<br>≥18 years were invited to participate in this study.<br>Randomization was implemented in a 2-step process. Initially,<br>participants were assigned to 1 of the 2 study groups<br>(appendicitis or severe acute malnutrition [SAM]) based on<br>their study ID. Subsequently, within each study group,<br>participants were stratified according to their academic year"		
**9) CONSORT: Mechanism used to implement the random allocation sequence (such as sequentially numbered containers), describing any steps taken to conceal the sequence until interventions were assigned**		
As the allocation to any study group was shortly followed by the study itself (within few minutes) there were no further steps taken to conceal the sequence before interventions were assigned.		
**10) CONSORT: Who generated the random allocation sequence, who enrolled participants, and who assigned participants to interventions**		
"To maintain the integrity of the study, the data analysis team remained<br>independent of the data collection process."<br><br>The allocation sequence was generated by the developers of the study: the first four authors mentioned in the article.		
**11a) CONSORT: Blinding - If done, who was blinded after assignment to interventions (for example, participants, care providers, those assessing outcomes) and how**		
**11a-i) Specify who was blinded, and who wasn't**		
"To maintain the integrity of the study, the data analysis team remained<br>independent of the data collection process."		
**11a-ii) Discuss e.g., whether participants knew which intervention was the "intervention of interest" and which one was the "comparator"**		
**11b) CONSORT: If relevant, description of the similarity of interventions**		
As this was a study focusing on medical education and elearning subitem 11b is not applicable to this trial.		
**12a) CONSORT: Statistical methods used to compare groups for primary and secondary outcomes**		

"We applied the same approach described in the previous point to evaluate the differences in postintervention knowledge levels across the groups. This analysis aimed to identify whether there were any significant differences in the knowledge levels between the groups after exposure to different learning resources, which could indicate the relative effectiveness of each resource"		
**12a-i) Imputation techniques to deal with attrition / missing values**		
all participants used the resources adaquately. There was no participant who dropped out.		
**12b) CONSORT: Methods for additional analyses, such as subgroup analyses and adjusted analyses**		
"To assess within-group knowledge acquisition, we compared the pre- and posttest results for each group. We used a Wilcoxon rank test for the 3 groups using textbook contents, e-learning materials, and self-guided internet materials. For the group using VP, we used a t test as the data were normally distributed. This analysis helped determine the extent of knowledge gain within each group after using their assigned learning resource"		
RESULTS		
**13a) CONSORT: For each group, the numbers of participants who were randomly assigned, received intended treatment, and were analysed for the primary outcome**		
this is shown in the consort flow diagram		
**13b) CONSORT: For each group, losses and exclusions after randomisation, together with reasons**		
this is shown in the consort flow diagram		
**13b-i) Attrition diagram**		
**14a) CONSORT: Dates defining the periods of recruitment and follow-up**		
"The study participants were recruited on November 29, 2021, and the study took place on December 10, 2021, at the main campus of LMMU in Lusaka, Zambia."		
**14a-i) Indicate if critical “secular events” fell into the study period**		
"Third, owing to a 1-hour delay at the study’s onset caused by technical issues, some participants might have experienced time pressure during the later stages (posttest and acceptance questionnaire), potentially introducing bias"		
**14b) CONSORT: Why the trial ended or was stopped (early)**		
The trial was not stopped early.		
**15) CONSORT: A table showing baseline demographic and clinical characteristics for each group**		
The article already describes the participants. Each group is described more closely in a table shown in the article.		
**15-i) Report demographics associated with digital divide issues**		
"Another aspect to consider is the familiarity with learning resources. Students might be more familiar with traditional learning resources, such as textbooks, compared with newer methods, such as VPs or e-learning platforms. This familiarity could influence the ease with which students can use and learn from these resources, thus affecting their knowledge acquisition."		
**16a) CONSORT: For each group, number of participants (denominator) included in each analysis and whether the analysis was by original assigned groups**		
**16-i) Report multiple “denominators” and provide definitions**		
Denominators are descriebed in a table in the results section		
**16-ii) Primary analysis should be intent-to-treat**		
**17a) CONSORT: For each primary and secondary outcome, results for each group, and the estimated effect size and its precision (such as 95% confidence interval)**		
The results are described in a table in the results section as well as a detailed description provided in the results section.		
**17a-i) Presentation of process outcomes such as metrics of use and intensity of use**		
**17b) CONSORT: For binary outcomes, presentation of both absolute and relative effect sizes is recommended**		
this is shown in the table in the results section.		
**18) CONSORT: Results of any other analyses performed, including subgroup analyses and adjusted analyses, distinguishing pre-specified from exploratory**		
No further subgroup analyses was performed further than the analysis to asses the primary outcomes.		
**18-i) Subgroup analysis of comparing only users**		
**19) CONSORT: All important harms or unintended effects in each group**		
No harms or unintended effects were reported.		
**19-i) Include privacy breaches, technical problems**		
Technical issues lead to a one-hour delay in performing the study. this is discussed more closely in the discussion section.		
**19-ii) Include qualitative feedback from participants or observations from staff/researchers**		
DISCUSSION		
**20) CONSORT: Trial limitations, addressing sources of potential bias, imprecision, multiplicity of analyses**		
**20-i) Typical limitations in ehealth trials**		
"Fifth, in our study, we acknowledge the possibility that participants may have been hesitant to provide negative feedback because of concerns regarding anonymity and potential implications for their academic performance"		
**21) CONSORT: Generalisability (external validity, applicability) of the trial findings**		
**21-i) Generalizability to other populations**		
"First, the generalizability of our findings is limited because of the study population, which exclusively consisted of third- and fourth-year BSc clinical science students. Consequently, our results may not be directly applicable to other contexts, such as different disciplines"		
**21-ii) Discuss if there were elements in the RCT that would be different in a routine application setting**		
**22) CONSORT: Interpretation consistent with results, balancing benefits and harms, and considering other relevant evidence**		
**22-i) Restate study questions and summarize the answers suggested by the data, starting with primary outcomes and process outcomes (use)**		

"In our study, we developed and assessed 2 interactive VP medical topics focusing on SAM and appendicitis, aiming to evaluate their effectiveness and acceptance compared with other prevalent learning resources at LMMU. The efficacy of transferring knowledge to students, and the precise impact of certain VP features, had previously been ambiguous. Our study aimed to address these aspects.<br>The primary aim of this study was to evaluate the acceptance and knowledge acquisition of BSc clinical sciences students at LMMU when using VPs as a learning resource in comparison with textbooks, preselected e-learning materials, and self-guided internet materials. A key finding of this study was that all 4 learning resources demonstrated their effectiveness in promoting knowledge gain within the study setting. Furthermore, VPs were well received by the students and proved to be noninferior compared with the other 3 learning methods."		
**22-ii) Highlight unanswered new questions, suggest future research**		
"Nonetheless, further research on the acceptability and effectiveness of VPs is warranted, as the incorporation of additional VP medical topics into the blended learning program at LMMU for BSc clinical science students is planned"		
Other information		
**23) CONSORT: Registration number and name of trial registry**		
Pan African Clinical Trials Registry (PACTR) PACTR202211594568574;<br>https://pactr.samrc.ac.za/TrialDisplay.aspx?TrialID=20413		
**24) CONSORT: Where the full trial protocol can be accessed, if available**		
The full trial protocol is accesible in the multimedia appendix		
**25) CONSORT: Sources of funding and other support (such as supply of drugs), role of funders**		
"This work was supported by the Else Kröner-Fresenius-Stiftung (2019_HA25). For the publication fee, the authors acknowledge financial support from the Deutsche Forschungsgemeinschaft within the funding program “Open Access Publikationskosten” as well as from the Heidelberg University"		
**X26-i) Comment on ethics committee approval**		
"This study was approved by the Heidelberg University Hospital Ethical Committee on August 30, 2021 (S-685/2021) and the LMMU Research Ethics Committee on November 29, 2021 (LMMU-REC 00005/21)."		
**x26-ii) Outline informed consent procedures**		
"We informed all potential and selected participants about the study’s objectives and procedures as well as their right to withdraw at any time without consequences. Before participation, each individual provided written informed consent, ensuring their voluntary involvement in the study."		
**X26-iii) Safety and security procedures**		
"Fifth, in our study, we acknowledge the possibility that participants may have been hesitant to provide negative feedback because of concerns regarding anonymity and potential implications for their academic performance. To address this concern, we implemented several measures to ensure anonymity of the data collected. These measures included (1) emphasizing the confidentiality of the study in the information provided to the participants, both verbally and in written form; (2) assigning unique participant identification numbers, which were not linked to personal information, to protect the identity of the participants during data collection and analysis; (3) ensuring that the questionnaires were completed individually and without peer or instructor influence; and (4) storing the collected data securely and restricting access to only the researchers directly involved in the study."		
**X27-i) State the relation of the study team towards the system being evaluated**		
"Conflicts of Interest:<br>OC was working for AMBOSS during the study but he was not involved in the data analysis nor did his affiliation influence any study outcomes."		
