# Supplementary material for: Evaluating the Effectiveness of Interactive Virtual Patients for Medical Education in Zambia: Randomized Controlled Trial
Source: JMIR Med Educ. 2023 Jun 29;9:e43699. doi: 10.2196/43699 (PMC10501501; doi:10.2196/43699)

**Virtual Patient Case: Appendicitis**

In the following, screenshots of virtual patient case in sequence are presented.


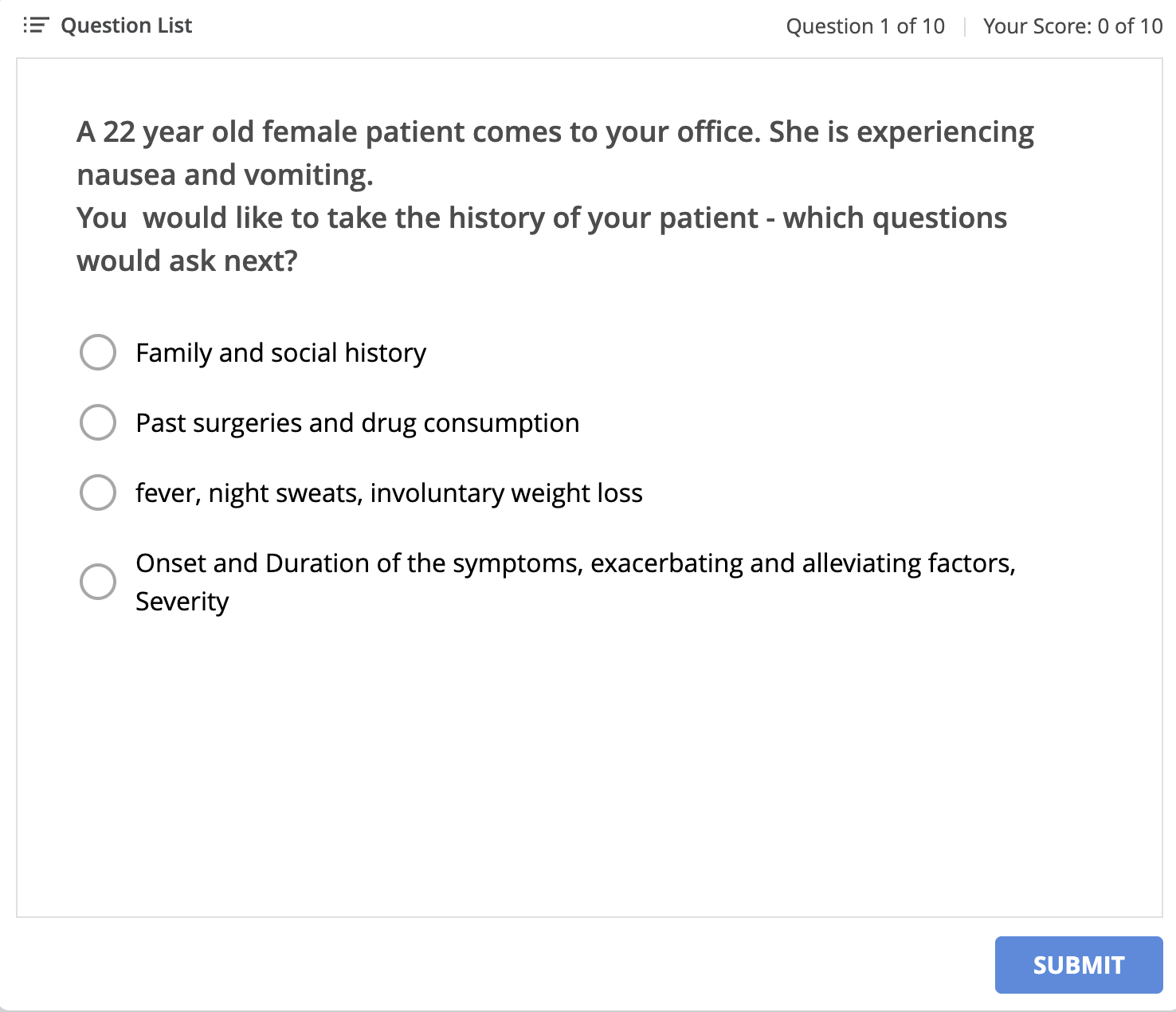


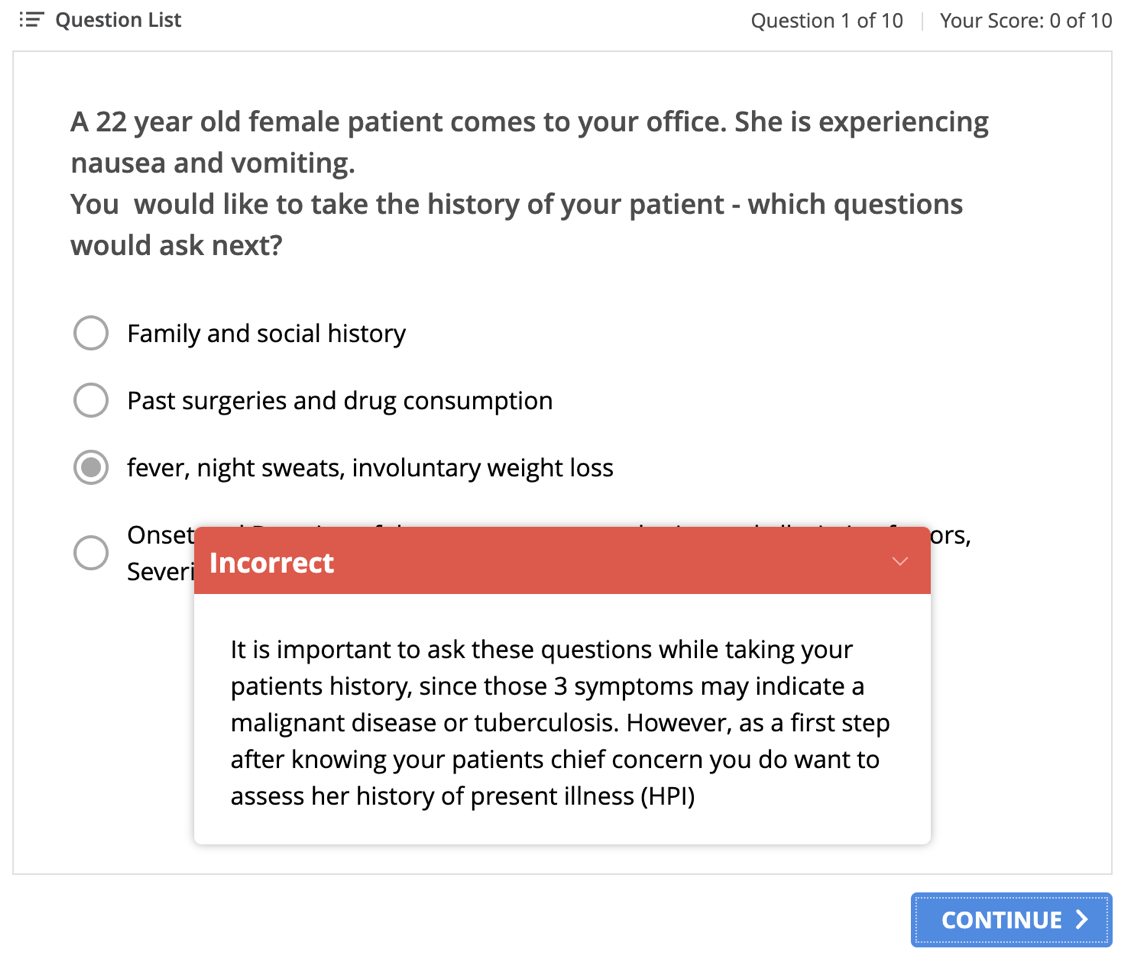


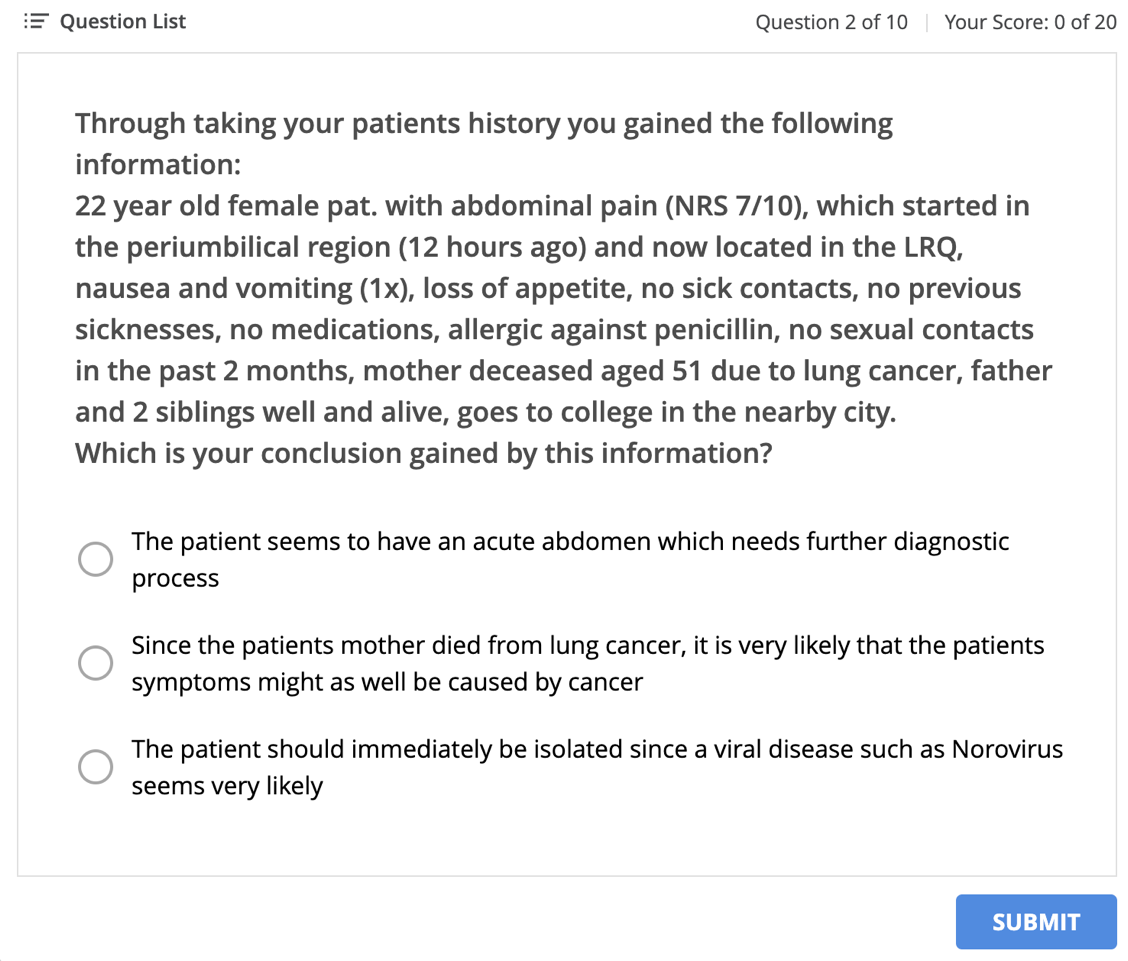


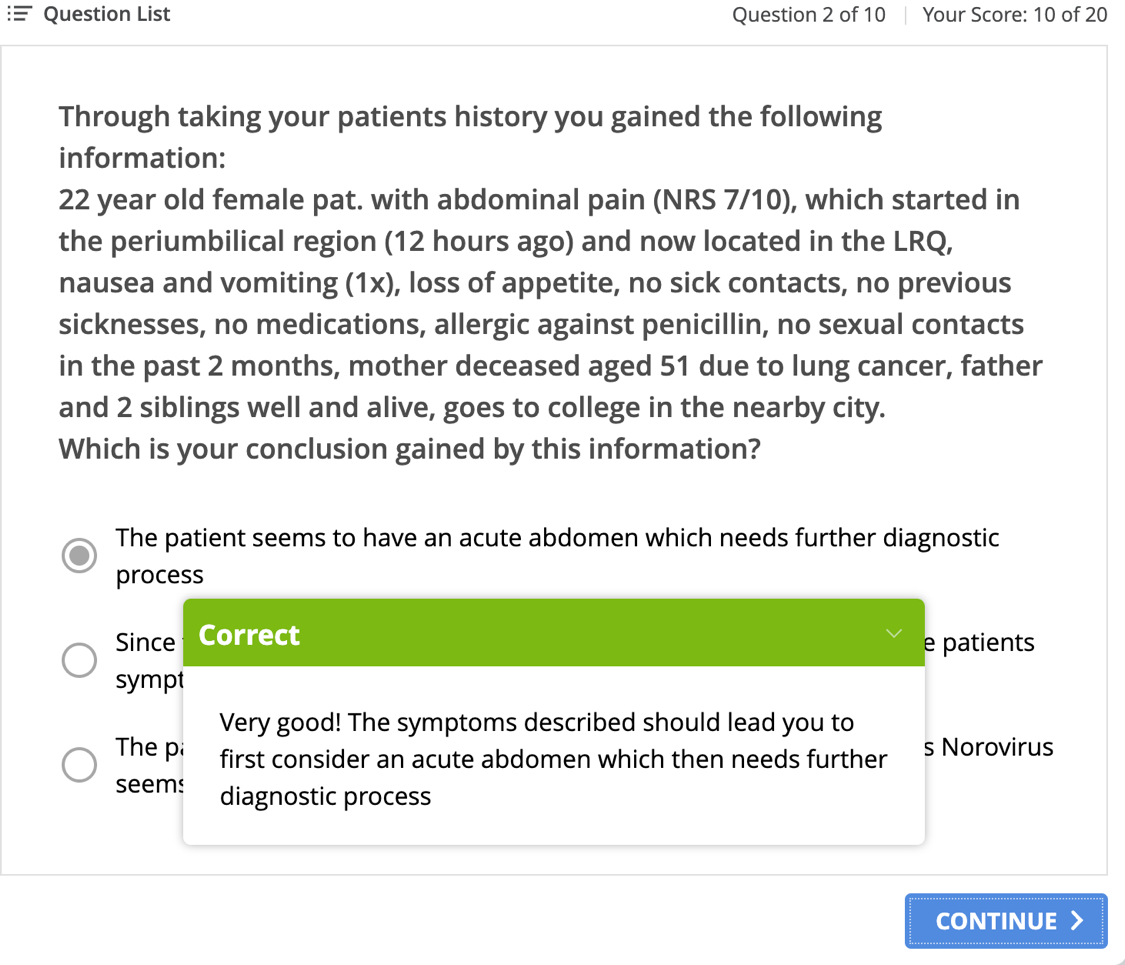


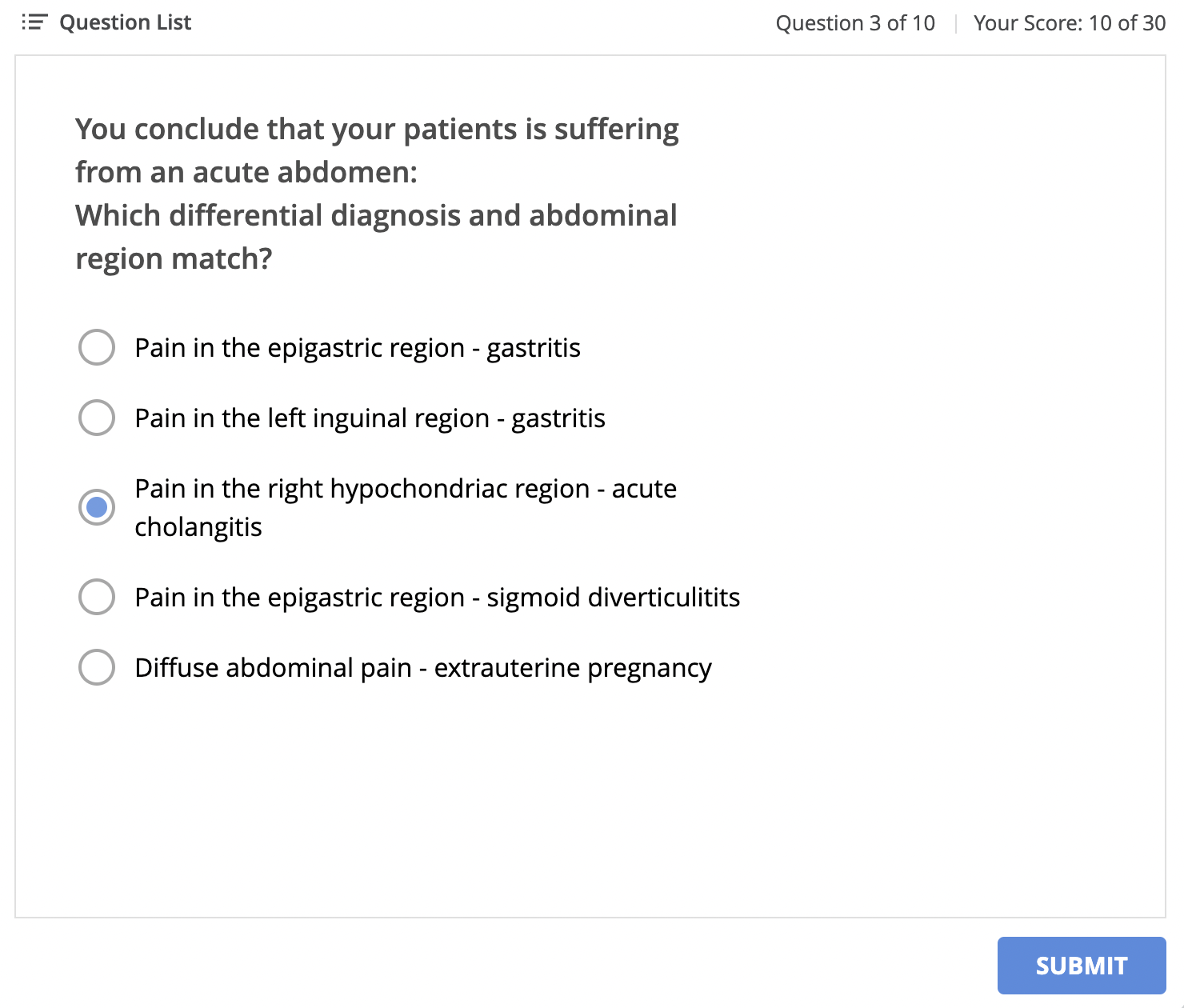


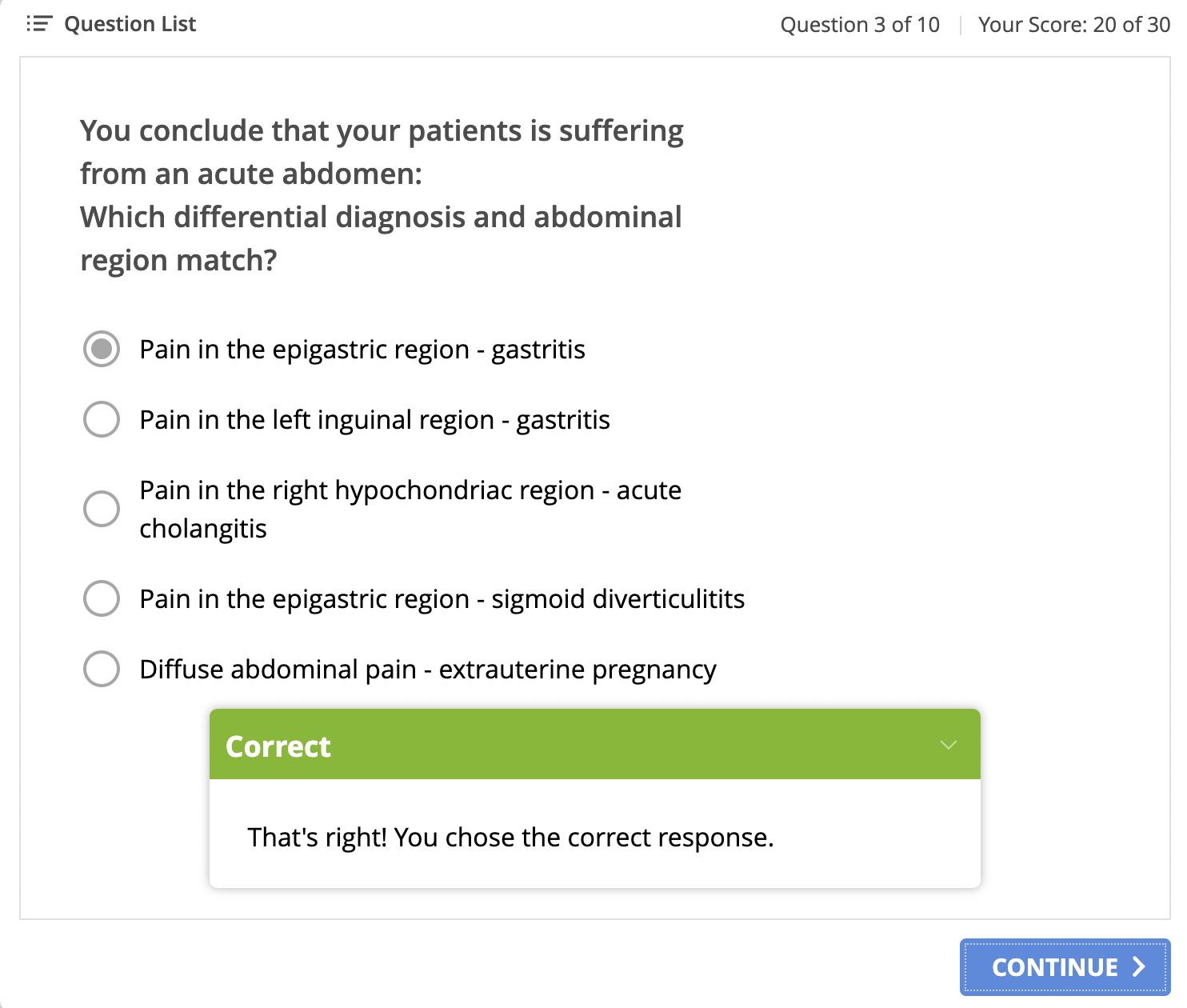


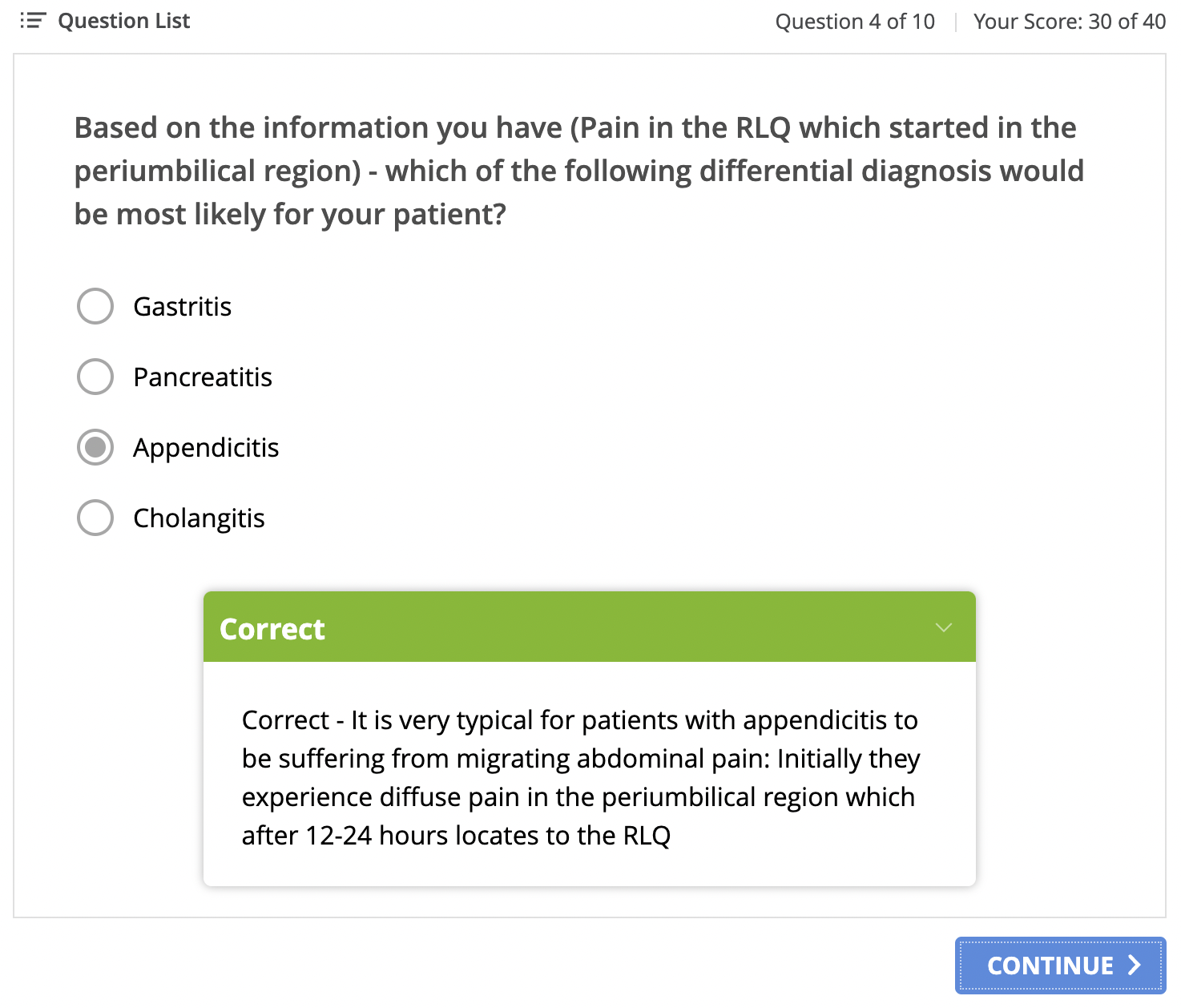


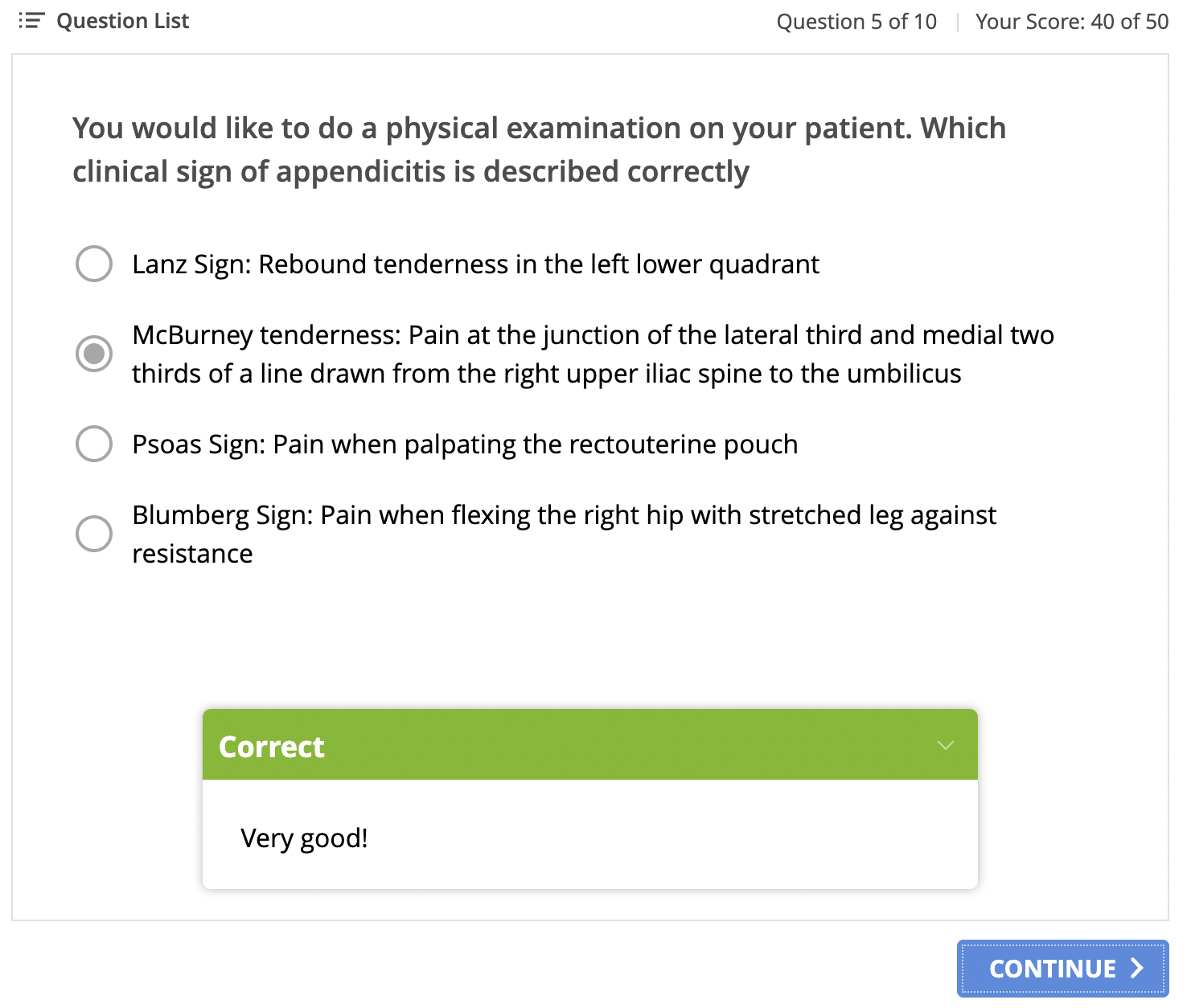


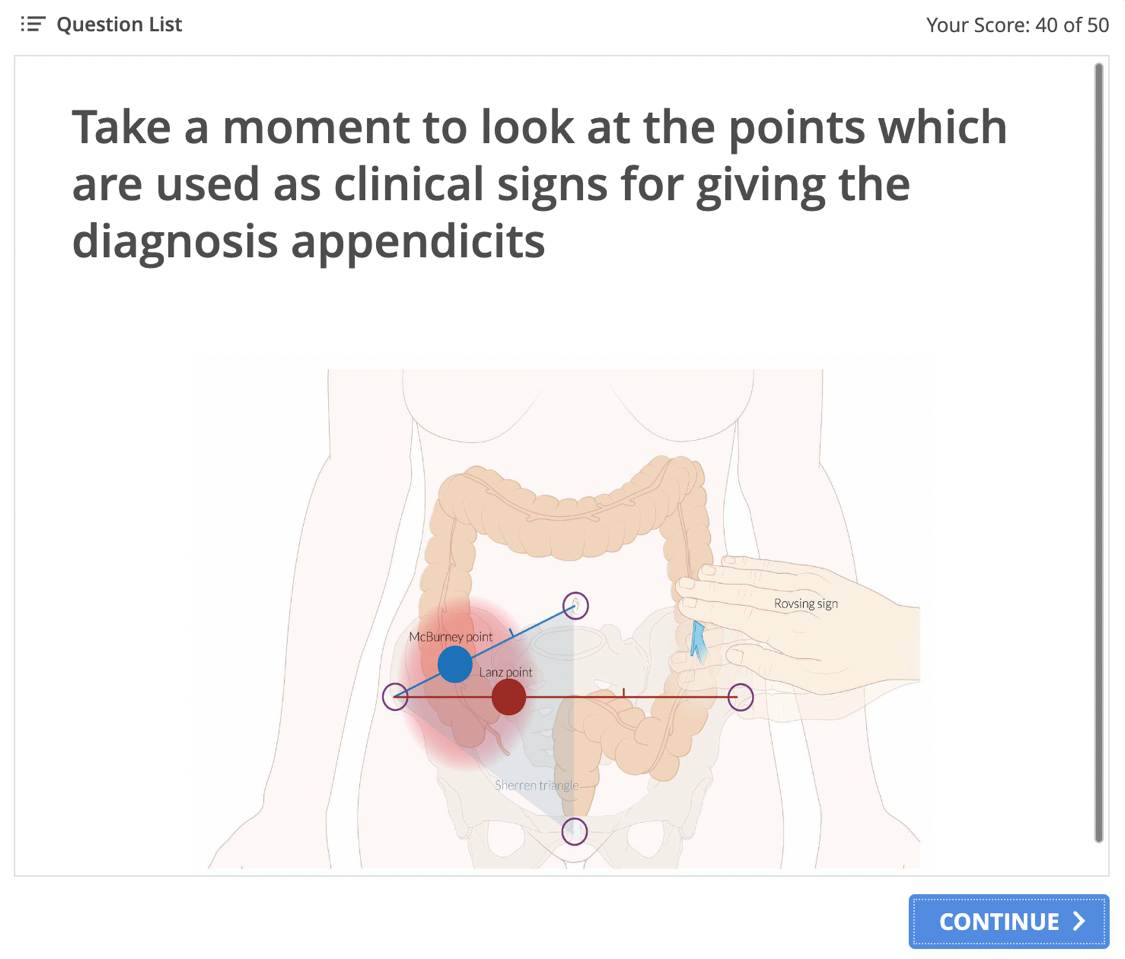


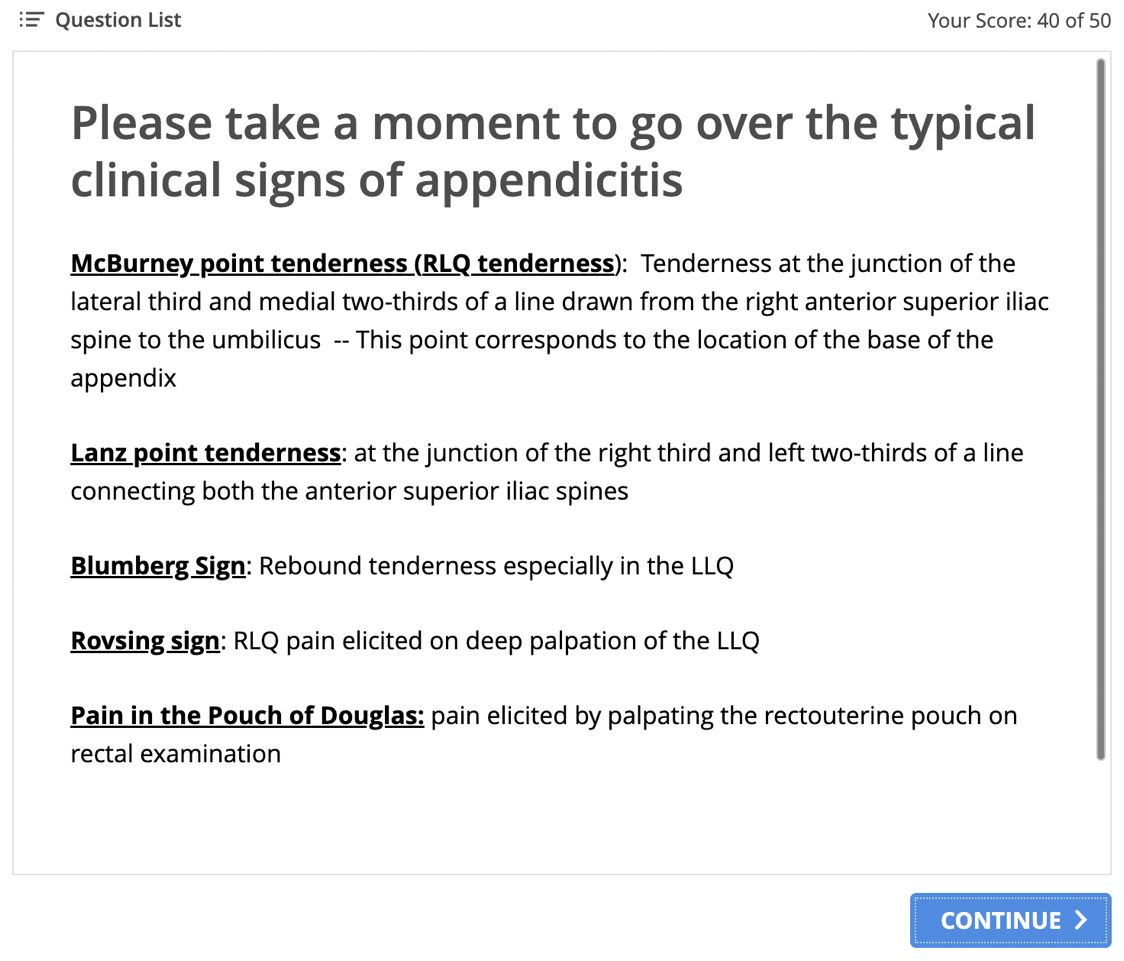


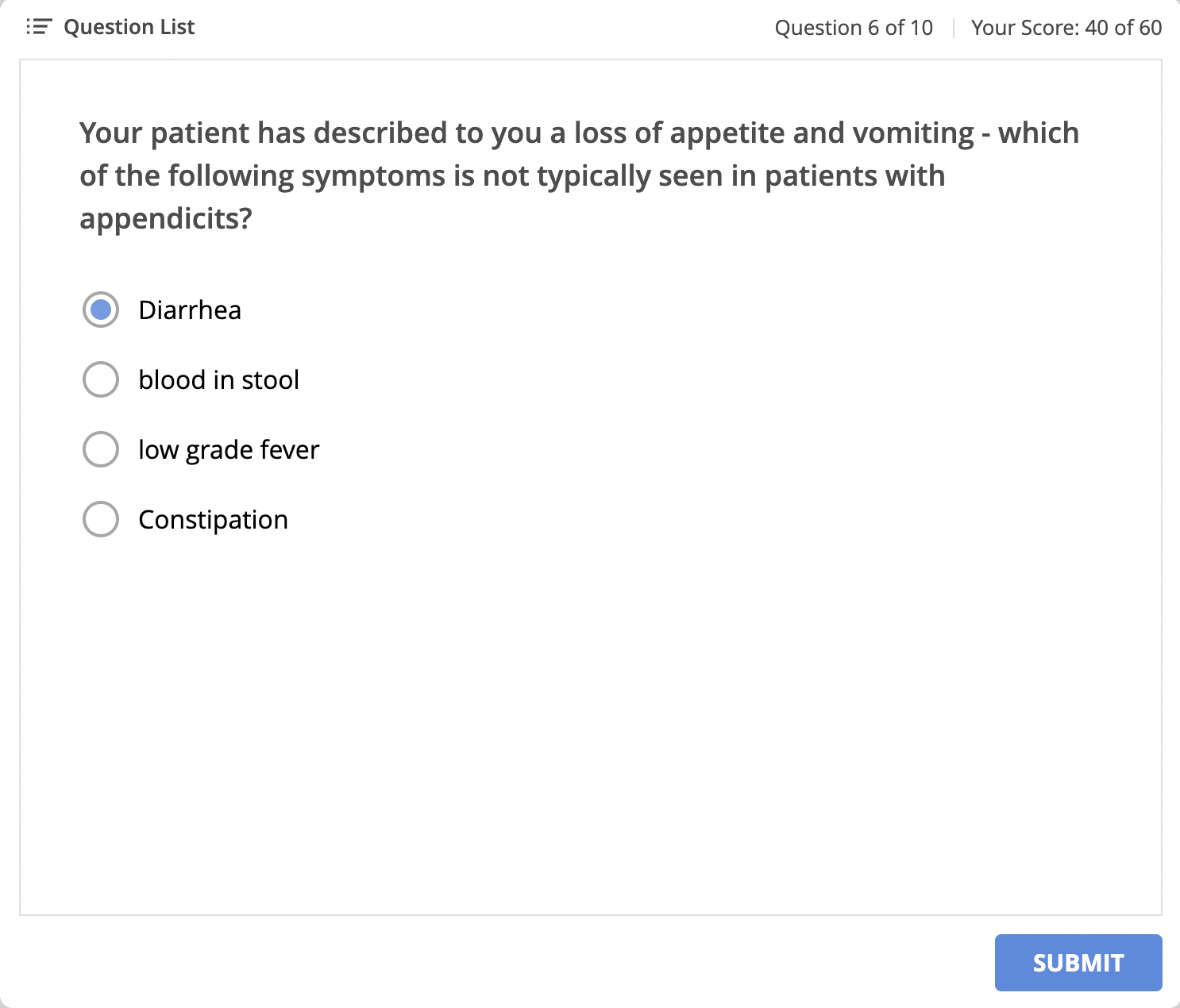


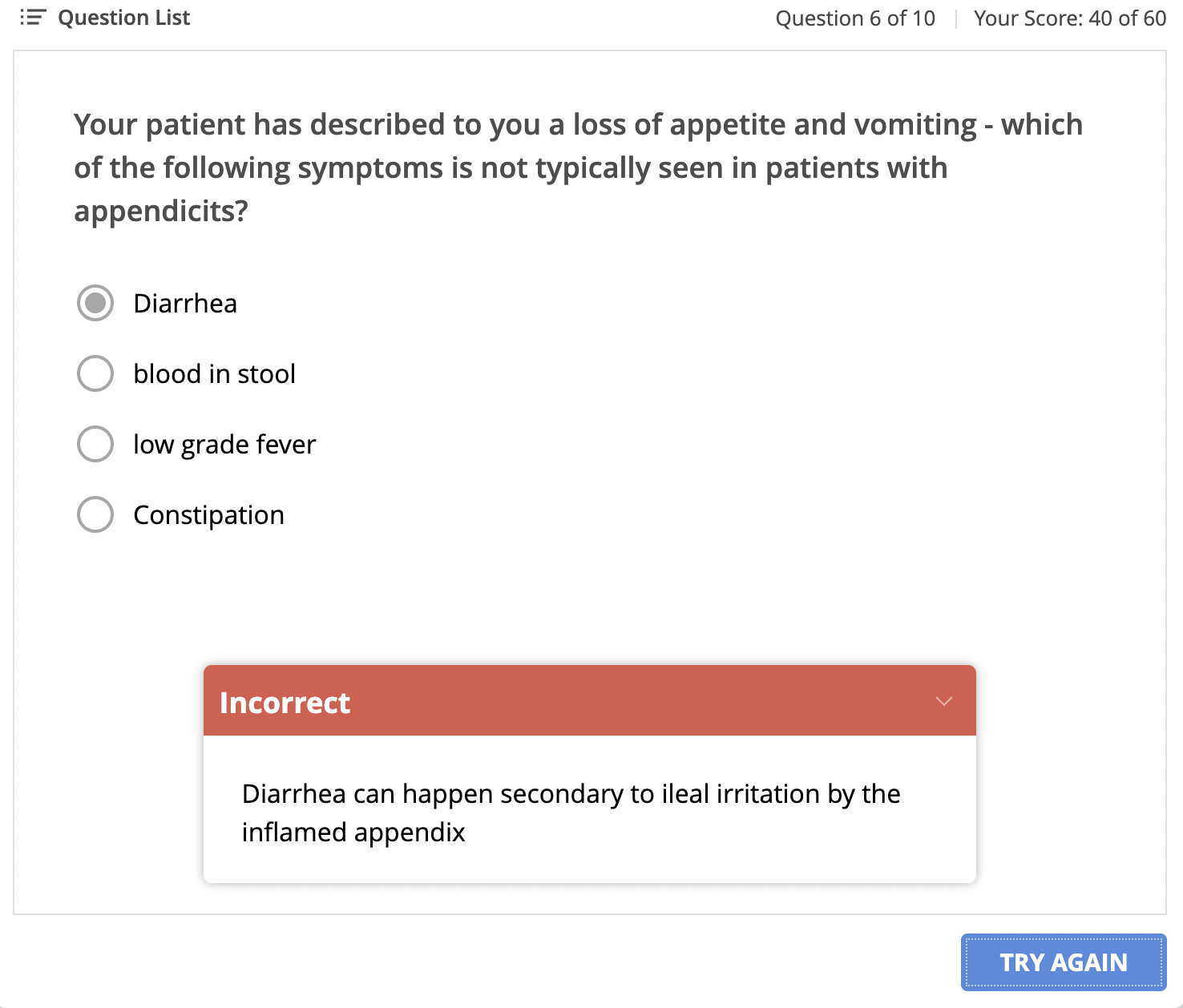


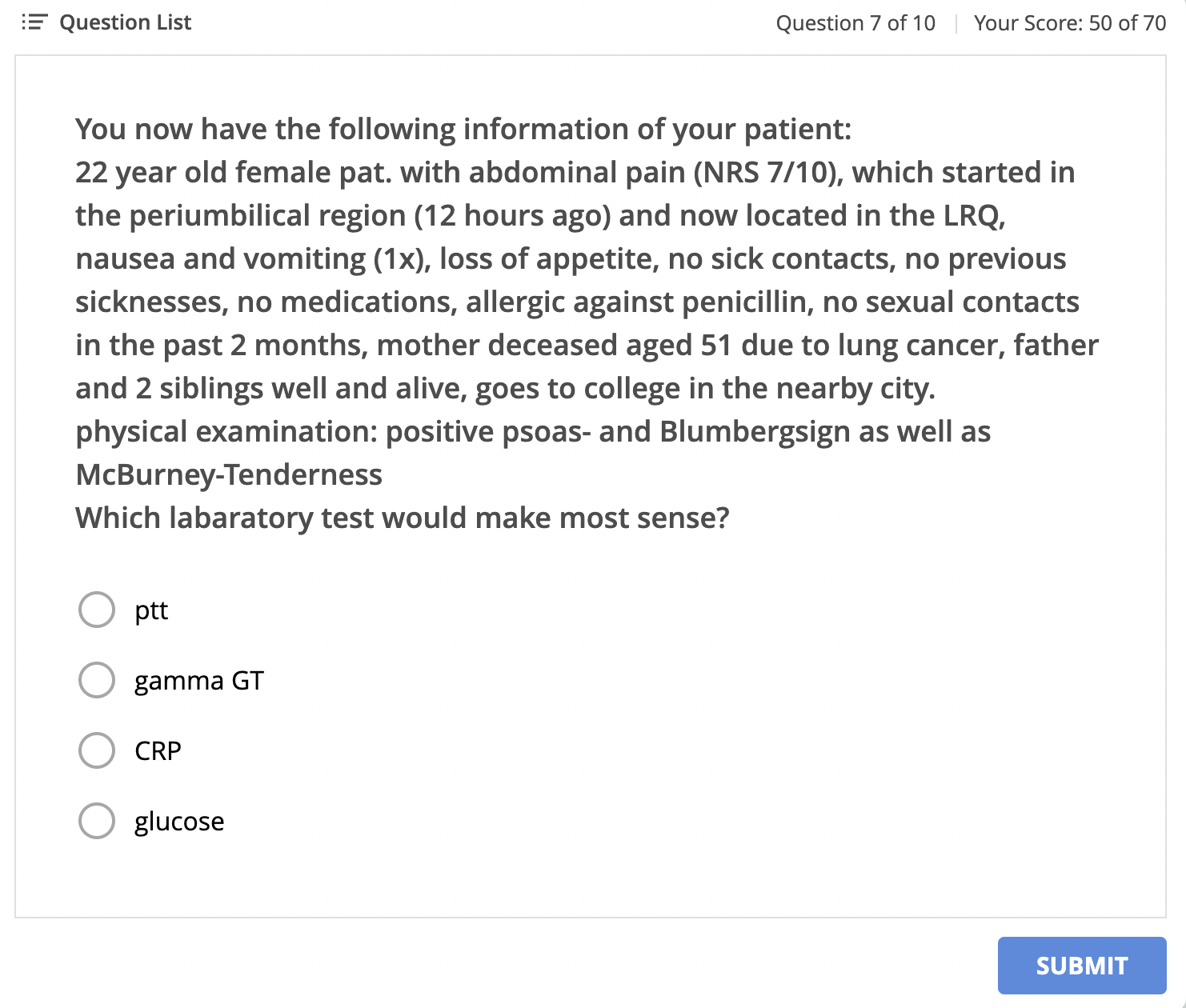


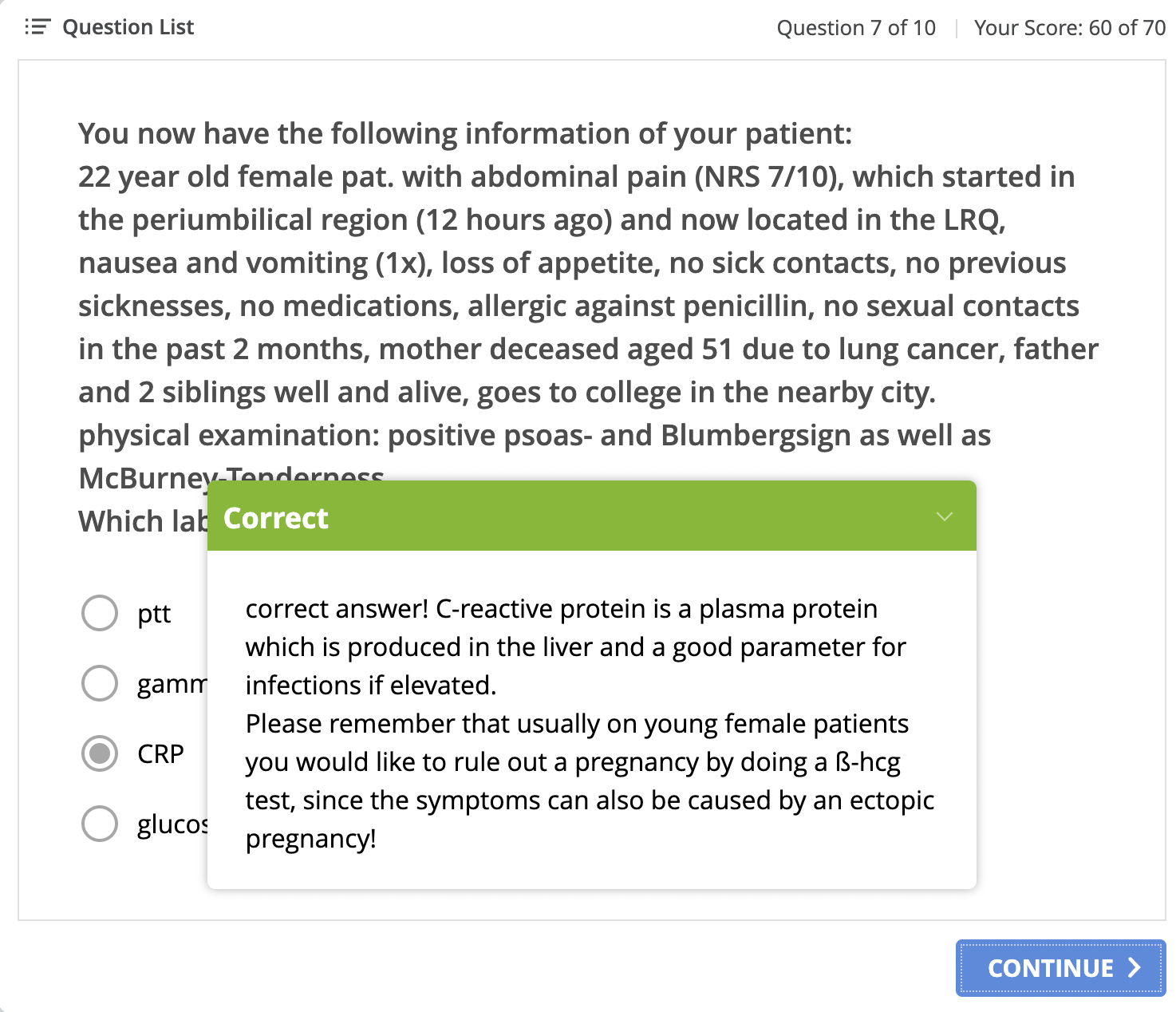


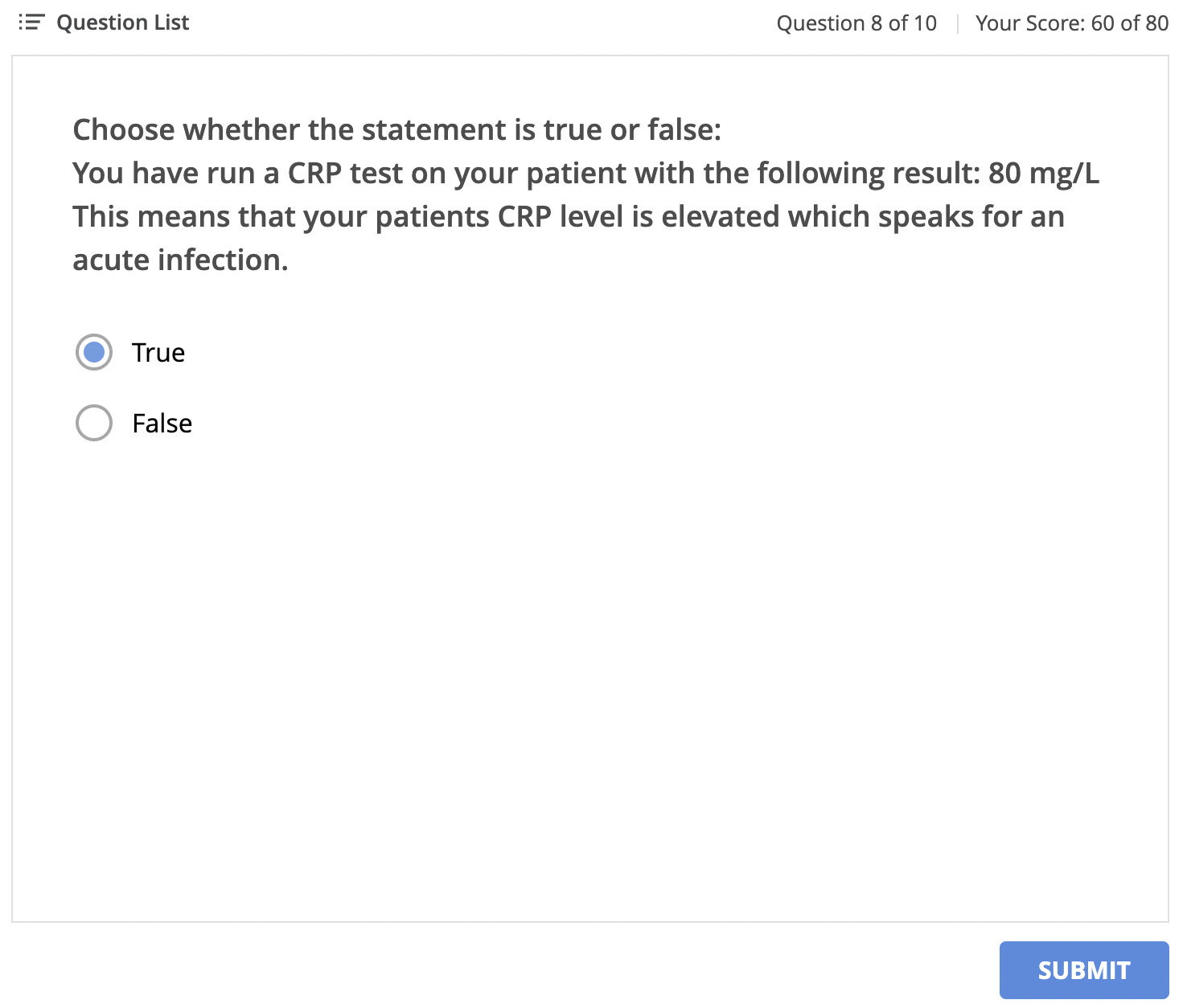


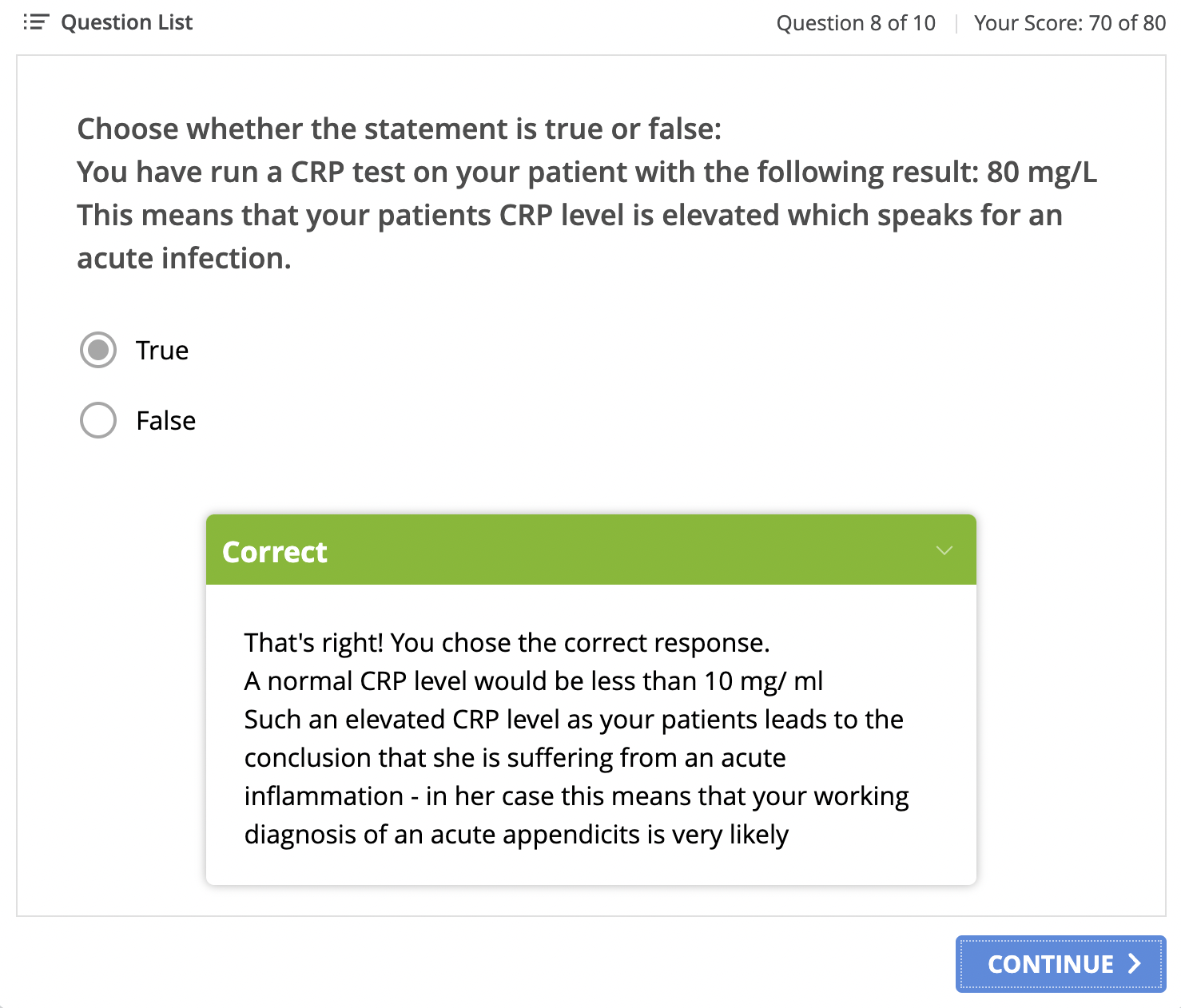


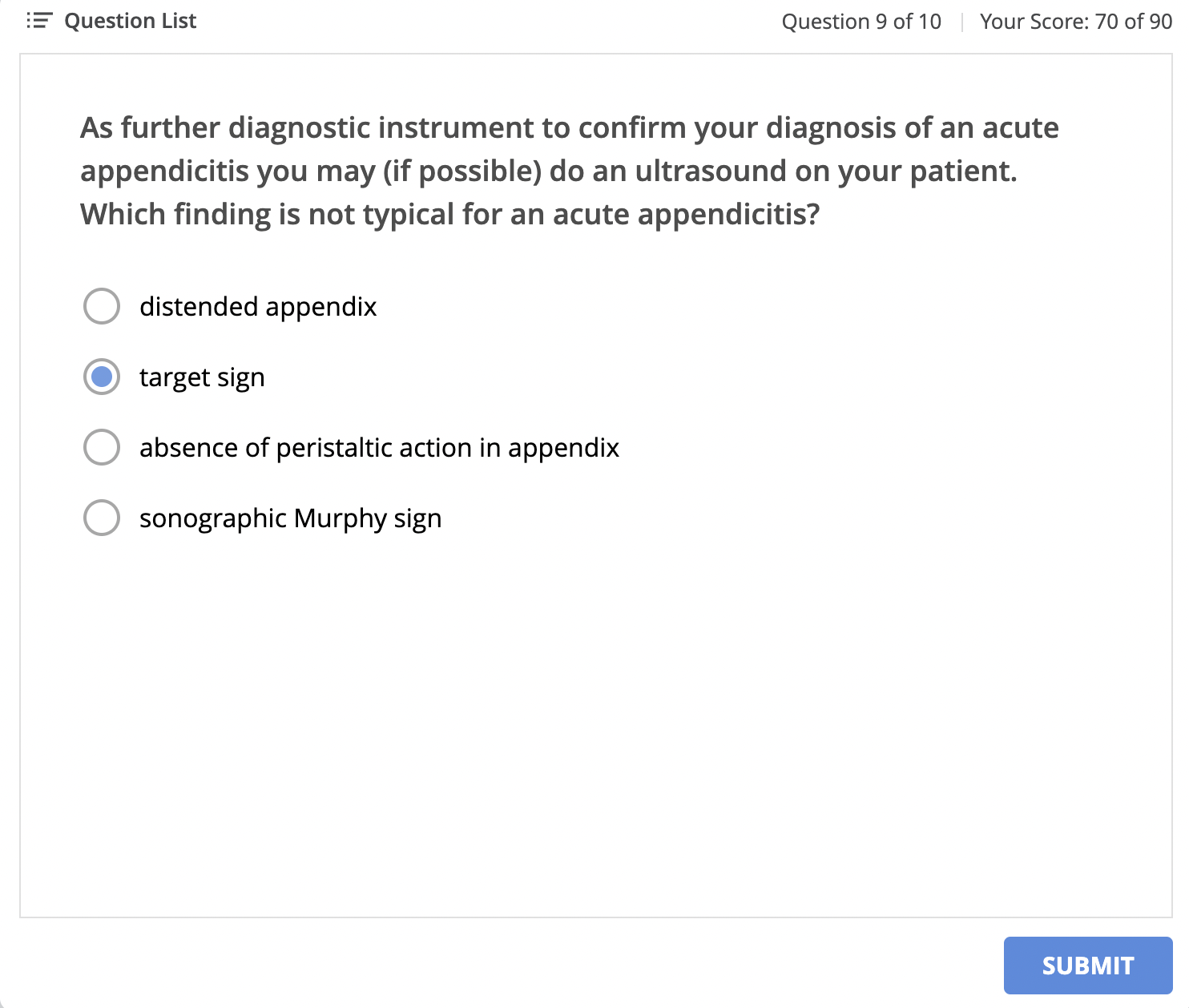


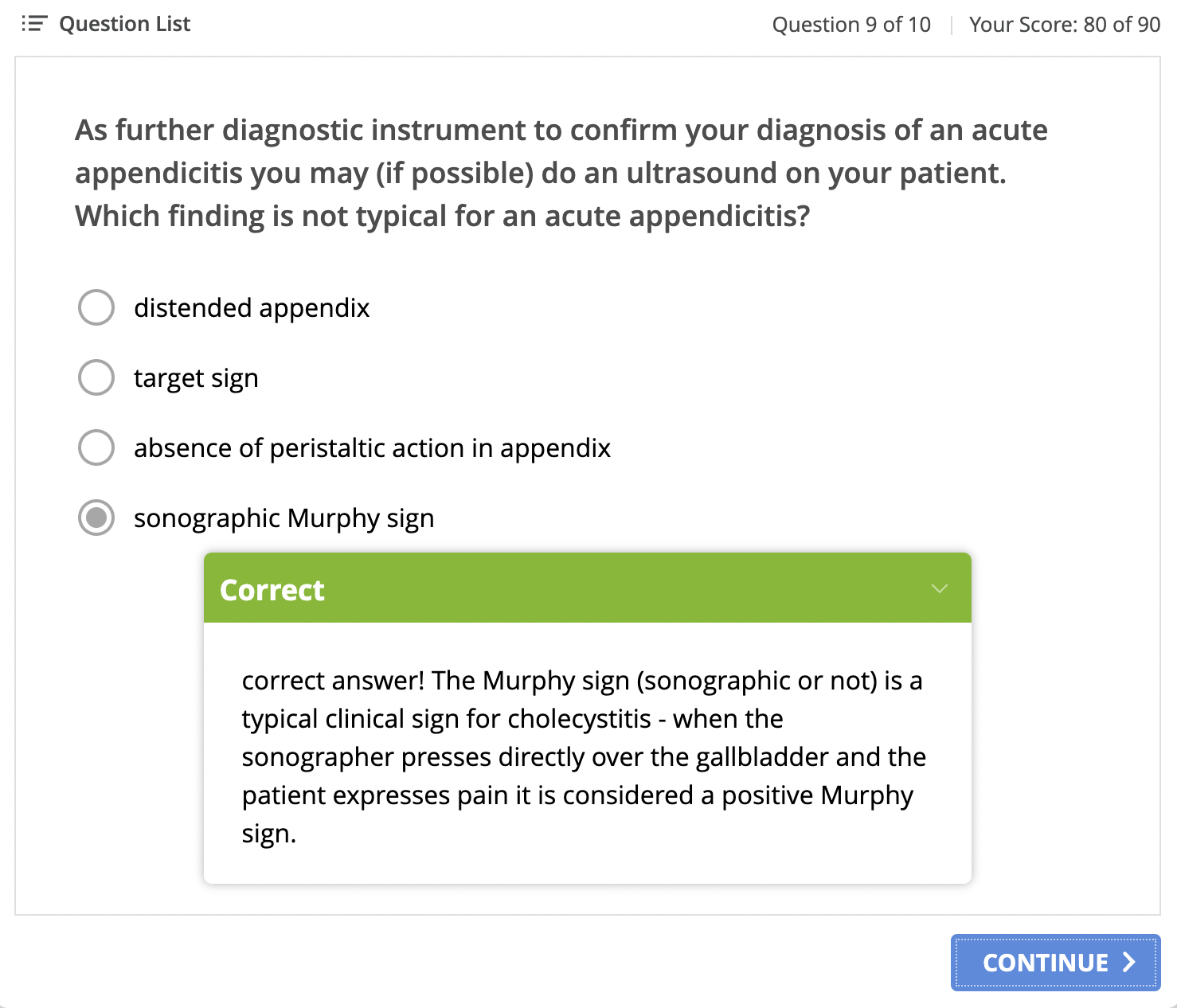


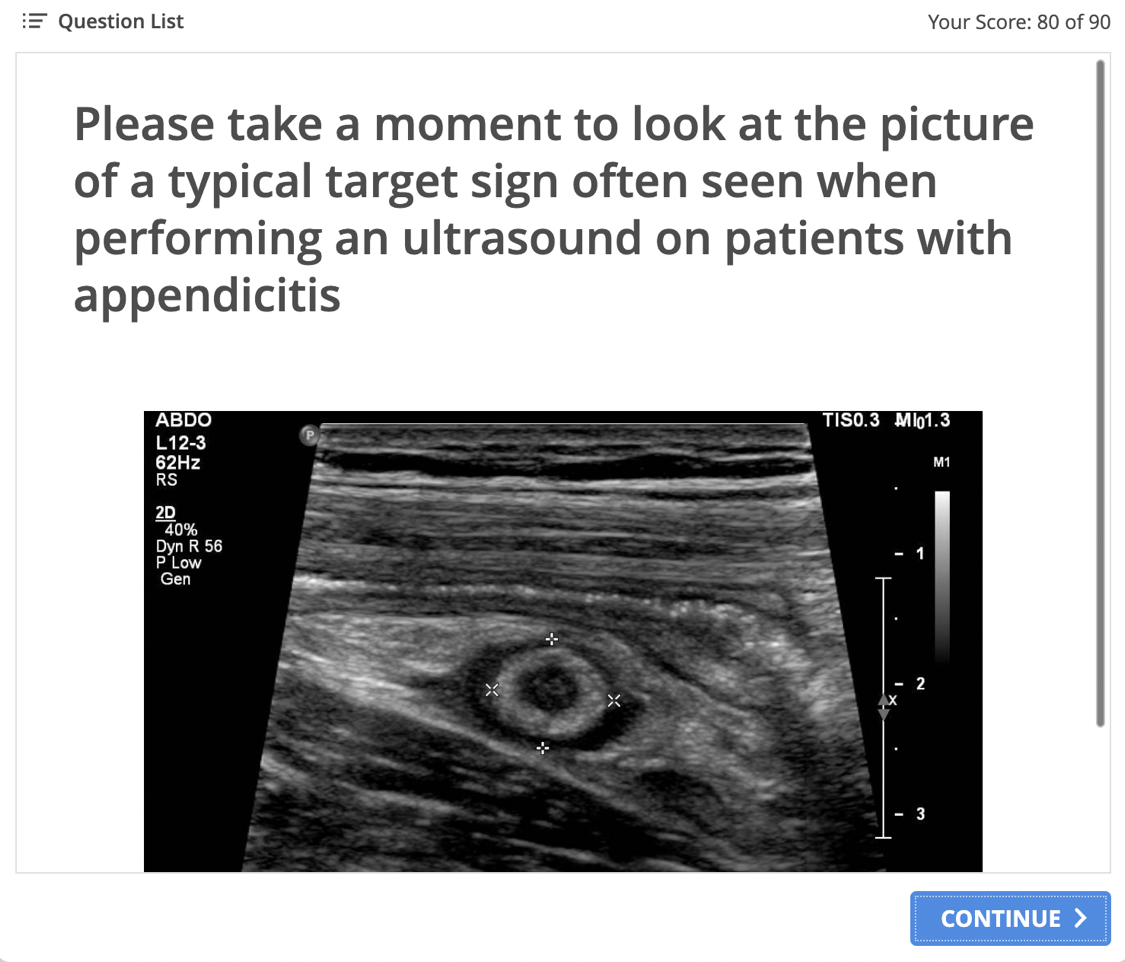


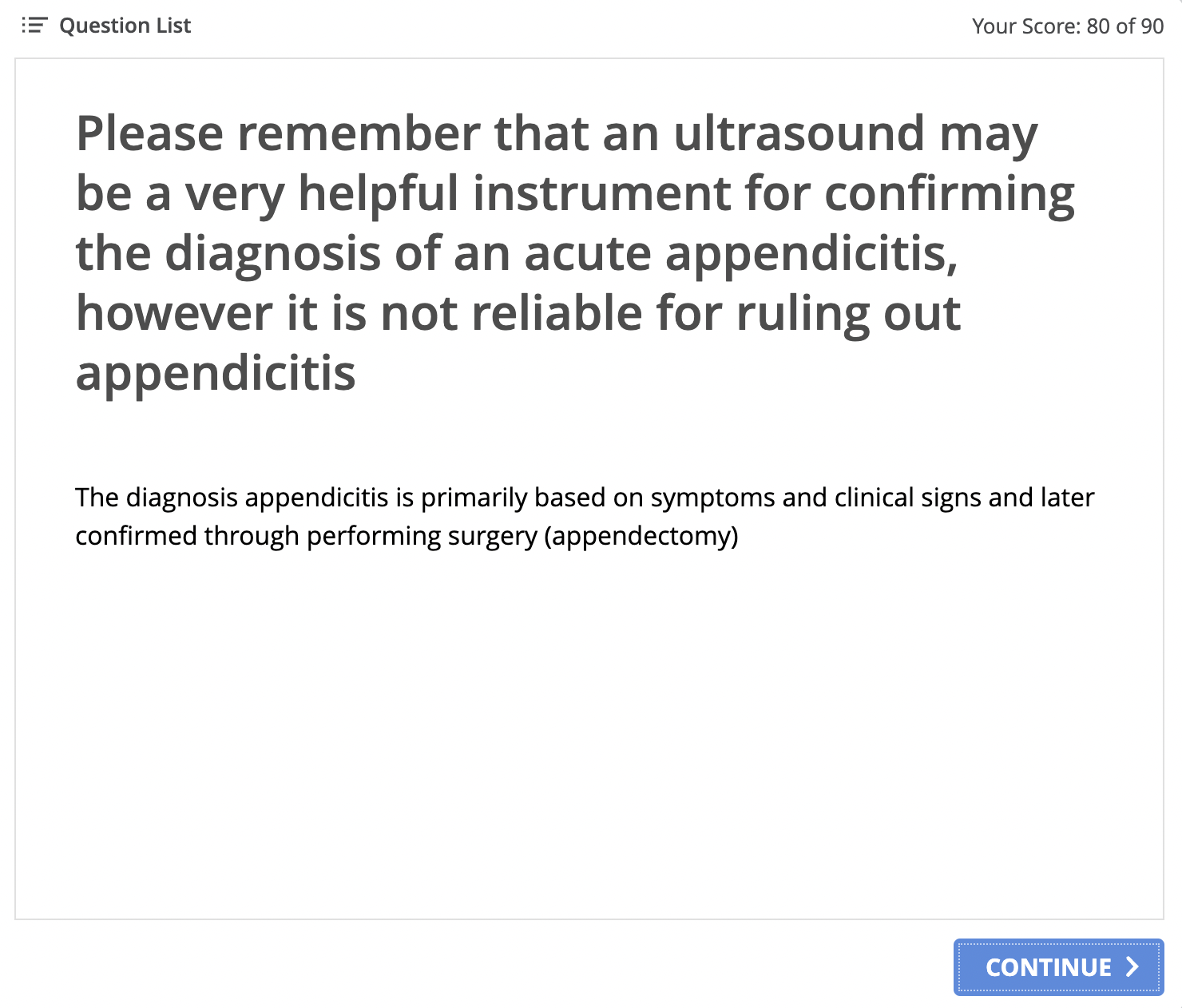


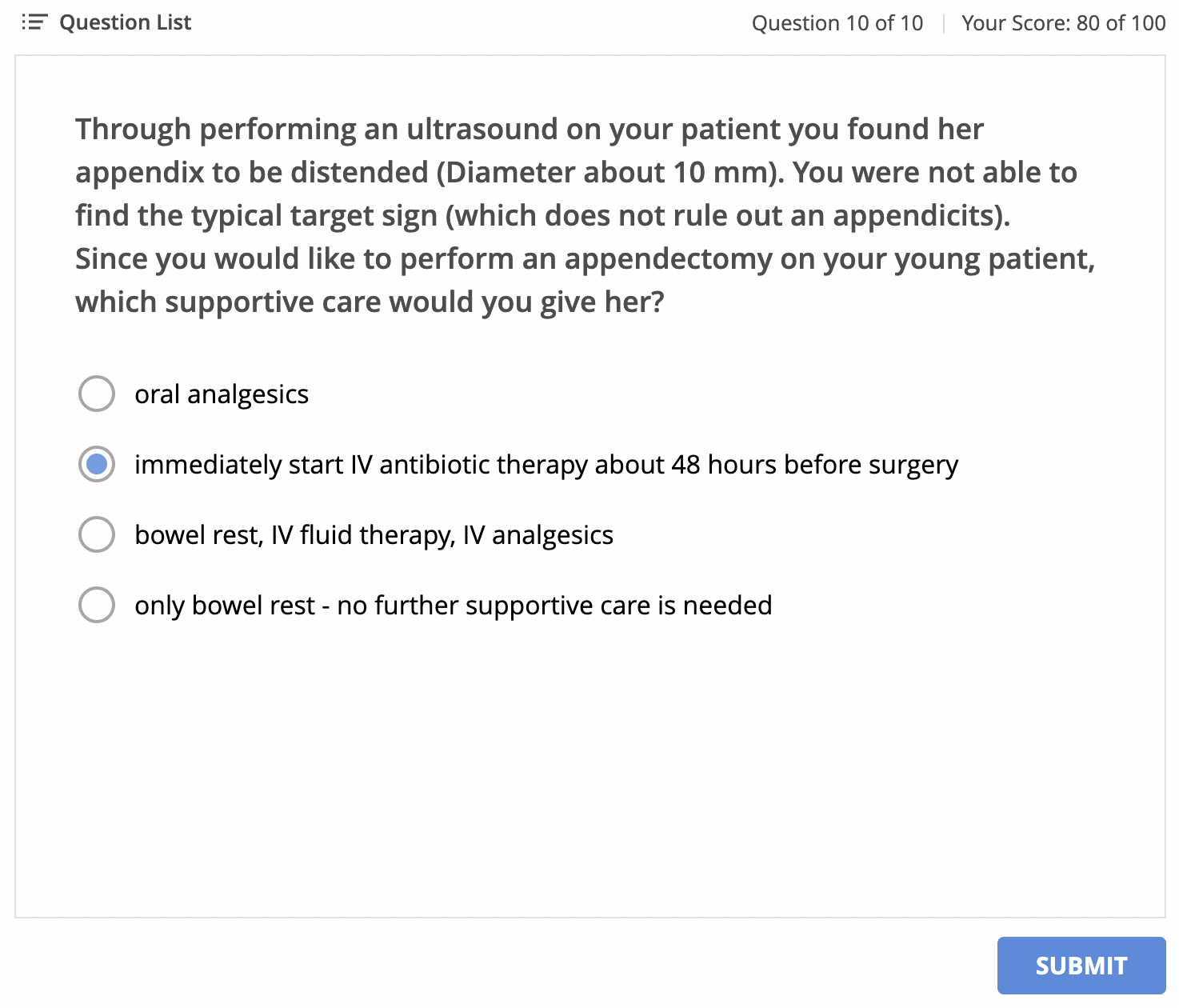


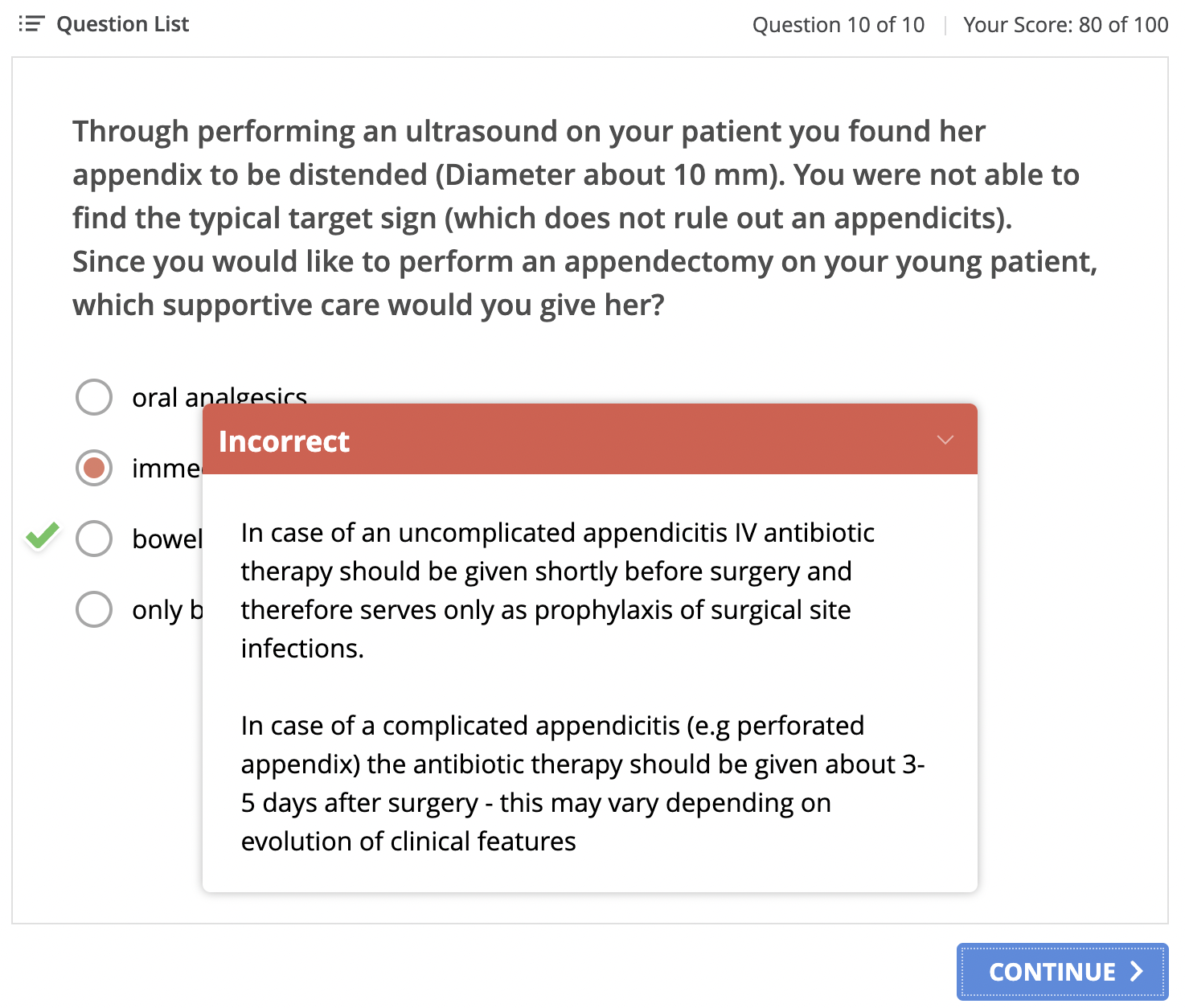


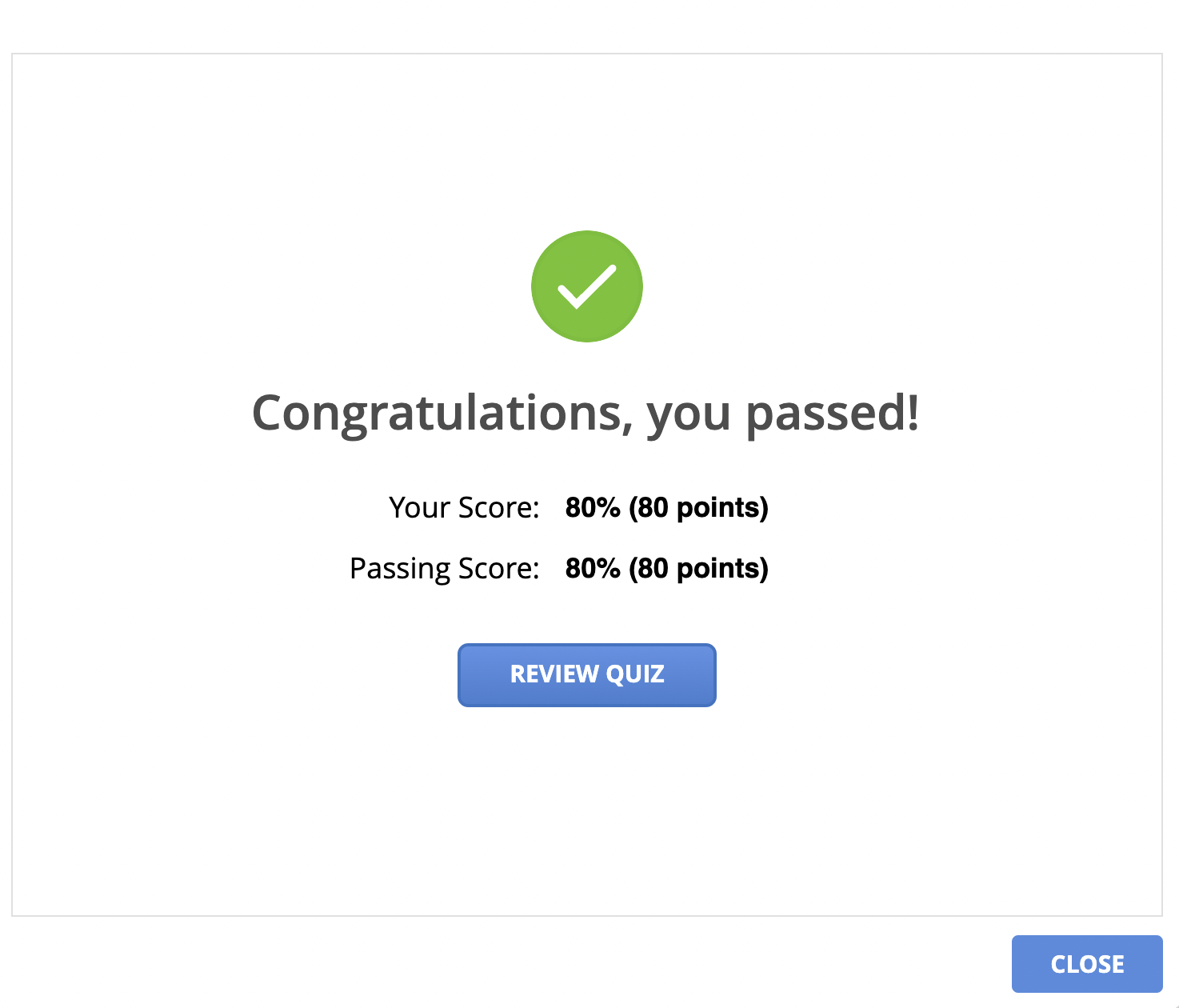

Supplement: Multimedia Appendix 3 [file mededu_v9i1e43699_app3.docx]
