# Supplementary material for: Evaluating the Effectiveness of Interactive Virtual Patients for Medical Education in Zambia: Randomized Controlled Trial
Source: JMIR Med Educ. 2023 Jun 29;9:e43699. doi: 10.2196/43699 (PMC10501501; doi:10.2196/43699)
Supplement: Multimedia Appendix 4 [file mededu_v9i1e43699_app4.pdf]

Attachments

1. Questionnaires

Demographic data

The following questions will be used to obtain information about the demographic data. These are completed along with the multiple-choice test and are placed before these knowledge questions.

How old are you? ____
What gender do you identify with? ◯ female ◯ male ◯ diverse
In which academic year are you? ◯ 3 ◯ 4

Questionnaire acceptance and self-perceived strengths/weaknesses

The questionnaire is based on the technology acceptance model (TAM), which originally includes five dimensions. To identify the reaction component four of the five dimensions are used (perceived usefulness, perceived ease of use, attitude towards using, behavioral intention to use). The fifth dimension (actual system use) is not used in this questionnaire because it isn´t relevant to our research question. (Davis 1989) Though, a fifth dimension (Job relevance) is added originating from an extended TAM named TAM 2 to access the behavior component (Venkatesh 2000). The five dimensions used to provide answers to the following five questions, which serve to answer the research question in all facets:

- **perceived usefulness**: To what extent do students believe that using the learning resources enhances their learning? (Davis 1989)
- **perceived ease of use**: To which degree do students believe that using the learning resources is free of effort? (Davis 1989)
- **Attitude towards using**: What is the attitude of students towards the learning resources? (Davis 1989)
- **behavioral intention to use**: To what extent does a student intend to use the learning resource? (Davis 1989)
- **job relevance:** To what extent do students believe that using learning resources enhances his/her work performance? (Venkatesh 2000)

Check the box with the answer that most applies to you.

1: strongly agree

2: agree

3: Neither agree nor disagree

4: disagree

5: strongly disagree

	1	2	3	4	5
**Perceived ease of use**					
I think using the learning resource is easy.					
Interacting with the learning resource requires a lot of effort					
**Perceived usefulness**					
I learned effectively using this learning resource.					
I feel like this learning resource is efficient.					
I think this learning resource is a good instrument to acquire knowledge.					
I think the learning resource is adequate for providing this content.					
**Attitude**					
I liked using this learning resource.					
I am positive towards using this learning resource.					
**Behavioral intention**					
If given the opportunity I would be a frequent user of this learning method.					
If given the opportunity I would favor this learning resource over others.					
I will recommend this learning resource to others.					
**Job Relevance**					
I think I will find it easy to apply the knowledge gained with this learning resource to a situation in the daily clinical practice.					
**Perceived enjoyment**					
I enjoyed using this learning resource to acquire this information.					
The use of the learning resource arouses my curiosity.					
I find this learning resource fun.					
