# Supplementary material for: Evaluating the Effectiveness of Interactive Virtual Patients for Medical Education in Zambia: Randomized Controlled Trial
Source: JMIR Med Educ. 2023 Jun 29;9:e43699. doi: 10.2196/43699 (PMC10501501; doi:10.2196/43699)
Supplement: Multimedia Appendix 5 [file mededu_v9i1e43699_app5.docx]

2. Pre & post-tests

a.) Severe Acute Malnutrition

- 1. 1. Severe acute malnutrition is defined by which of the following a) stunting
  2. b) wasting
  3. c) underweight
  4. d) age less than 5 years
  5. 2. Severe acute malnutrition is defined by the following except; a) Mid upper arm circumference less than 11.5cm
  6. b) weight for length Z score < -3
  7. c) bilateral pitting edema
  8. d) child aged 5 months with weighing 3.5kg
  9. 3. Underweight in children is defined by which of the following: a) Mid upper arm circumference less than 11.5cm
  10. b) weight for length Z score < -3
  11. c) weight for age z score <-3
  12. d) height for age z score < -3
  13. 4. Which of the following defines stunting in children a) Mid upper arm circumference less than 11.5cm
  14. b) weight for length z score < -3
  15. c) weight for age z score <-3
  16. d) height for age z score < -3
  17. 5. Which of the following are risk factors for developing malnutrition? a) having 3 meals per day
  18. b) having 3 children all below the age of 5
  19. c) history of diarrhea
  20. d) mother aged 25years
  21. 6. The following are complications of severe acute malnutrition except a) Hyperglycemia
  22. b) Hypothermia
  23. c) Sepsis
  24. d) Dehydration
  25. 7. The phases of treatment of malnutrition include all of the following except a) Stabilization
  26. b) Resuscitation
  27. c) Rehabilitation
  28. d) Follow up
  29. 8. The following are required to transition a patient from F75 to F100 or RUTF a) Walking
  30. b) Smiling
  31. c) Finishing feeds

1. d) Resolving edema
   1. 9. How does F75 differ from F100? a) F75 has more calories than F100
   2. b) F75 is used in the rehabilitation phase
   3. c) F75 has higher protein content
   4. d) F75 has more iron content
   5. 10. Which of the following patients should be considered for discharge from inpatient therapeu- tic program? a) A patient with edema 3+ but parent say the able to give RUTF at home
   6. b) Patient who finishes two thirds of the days ration of RUTF
   7. c) A patient with an absent caretaker who has not received nutritional counselling
   8. d) A patient with WLZ score <-1 and no complications
   9. 11. The treatment of Shock in malnourished children includes the following except; a) Bolus of Normal Saline over 20 minutes
   10. b) Active warming
   11. c) Correction of hypoglycemia
   12. d) Blood transfusion
   13. 12. Malnourished children with dehydration should receive rehydration therapy with; a) Resomal
   14. b) Ringer lactate
   15. c) Half strength Darrows
   16. d) Normal saline
   17. 13. Moderate malnutrition is defined as a) Mid upper arm circumference < 14mm
   18. b) Weight for length Z score: Median with bilateral pitting edema
   19. c) Weight of height Z score < -2
   20. d) Height for Age Z score < - 1
   21. 14. Oral Rehydration Solution (ORS) is used in SAM children with which of the following condi- tions; a) Dysentery
   22. b) Hypernatremic dehydration
   23. c) Cholera
   24. d) Typhoid
   25. 15. The treatment of hypothermia includes all of the following except; a) bathing children with hot water
   26. b) removal of the wet clothes off the children
   27. c) use of warm blankets and heater
   28. d) Closure of open windows
   29. 16. Concerning sepsis with of the following is not TRUE? a) IV antibiotics should be started promptly
   30. b) Blood culture is the gold standard for diagnosis
   31. c) Normal temperature reading excludes a diagnosis of sepsis
   32. d) Gastric lavage should be performed if the patient has signs and symptoms of pneumonia
   33. 17. 4-year-old Bramon is admitted to the malnutrition ward with history of watery diarrhea for 2 weeks. On examination he is severely wasted with bilateral edema 3+. You confirm watery stool on the ward and start rehydration therapy. Which of the following investigations would you order to aid in his management? a) Stool- Modified ZN stain
   34. b) Protein in stool
   35. c) Stool Microscopy
   36. d) Stool for reducing sugars
   37. 18. RUTF should be prescribed to which of the following patients; a) A patient with a failed appetite
   38. b) SAM without complications
   39. c) weight for age <-3
   40. d) moderate acute malnutrition
   41. 19. A 3year old is admitted to your health facility with severe acute malnutrition with pneumo- nia. She is placed on oxygen therapy and noted to be very ill; she weighs 4kg and her WLZ score is -4. The working physician her prescribes F100 feeds every 3 hours and intravenous antibiotics. The next day you are called to review the patient because she is in severe respira- tory distress, her weight now 5kg and she has bilateral edema. What is the most likely reason for her signs and symptoms. a) Improving weight due to feeding every 3 hours
   42. b) allergic reaction to F100
   43. c) worsening malnutrition
   44. d) Refeeding feeding
   45. 20. Dermatosis seen in malnutrition is due to deficiency of which of the following nutrients? a) Zinc
   46. b) selenium
   47. c) magnesium
   48. d) iron
   49. 21. Concerning refeeding syndrome, which of the following electrolyte abnormalities are com- mon? a) Hyponatremia
   50. b) Hyperphosphatemia
   51. c) Hypokalemia
   52. d) Hypermagnesemia

b.) Appendicitis

**1. Which of the following answers concerning medical history taking is wrong?**

1. a) The objective is to develop a good physician-patient relationship and figure out a differential diagnosis
2. b) If possible, the setting is a quiet room without other patients around
3. c) A review of all organ systems should always be taken
4. d) Medical history taking includes description of chief concern, history of present illness, past medical history, family history and social history

**2. Which answer concerning the epidemiology of appendicitis is wrong?**

1. a) Appendicitis is a very common cause for acute abdomen
2. b) Appendicitis is very common amongst young patients
3. c) Appendicitis is more common in women/girls than in men/boys
4. d) The peak incidence of appendicitis is between 10-19 years

**3. A very common and specific symptom of appendicitis is the migrating abdominal pain – which answer is correct?**

1. a) Initially most patients experience diffuse pain in the epigastric region which then migrates to the umbilical region
2. b) Initially all patients experience pain in the right lower quadrant which migrates to the left lower quadrant
3. c) Initially patients experience very sharp pain in the umbilical region which becomes a more diffuse pain within the first 12 hours
4. d) Many patients initially experience diffuse pain in the umbilical region which migrates to the right lower quadrant

**4. Which of the following nonspecific symptoms is not typically seen in patients with appen- dicitis?**

1. a) Nausea
2. b) Rectal bleeding
3. c) Anorexia
4. d) Low grade fever

**5. Which of the following clinical signs of appendicitis is described correctly?**

1. a) McBurney point tenderness: Tenderness at the junction of the lateral third and medial two- thirds of a line drawn from the right anterior superior iliac spine to the umbilicus
2. b) Psoas-Sign: Rebound tenderness in the right lower quadrant
3. c) Lanz-Sign: Pain when flexing the right hip with stretched leg against resistance
4. d) Douglas-Pouch Pain: Tenderness in the epigastric region

**6. You have a young female patient which you believe might be having an appendicitis, which laboratory studies would make most sense? Study Protocol, ASA-Study – Version 1.2 (19th August, 2021)**

1. a) Bilirubin
2. b) Hemoglobin
3. c) CRP and ß-hcg
4. d) Troponin T

**7. Which supportive Therapy is most suitable for a patient with acute appendicitis?**

1. a) i.v. analgetics, bowel rest, i.v. fluids
2. b) oral analgetics, oral antibiotics,
3. c) placing patient on the left side to relieve the pain on the right side
   1. d) do an enema
4. **8. Which patients with acute appendicitis should receive antibiotic therapy?**
   1. a) Only the ones that develop a fever
   2. b) All patients except for pregnant women
   3. c) All patients should receive antibiotic therapy
   4. d) Only children and elderly patients

**9. Which therapy would be most suitable for patients with acute appendicitis?**

1. a) Conservative management with oral antibiotics is in most cases sufficient
2. b) Surgical appendectomy should be performed after 24 hours of antibiotic treatment
3. c) Surgical appendectomy should be performed only if the diagnosis of an acute appendicitis is confirmed through ultrasound
4. d) Surgical appendectomy should be performed as an emergency surgery if the diagnosis is highly likely

**10. Which answer for acute appendicitis in pregnant women and children is wrong?**

1. a) Pregnant women often present with pain in an atypical location (usually higher)
2. b) Children usually present with the same symptoms as adult patients
3. c) Ultrasound is the diagnostic procedure of choice when it comes to pregnant women and chil- dren
4. d) Perforated appendix in pregnant women is associated with a higher risk of fetal loss

**11. Concerning typical signs and symptom of acute appendicitis ,which of the following is wrong**

1. a. Fever
2. b. Hematuria
3. c. Right lower quadrant pain.
4. d. Nausea

**12. Concerning the etiology of acute appendicitis, which of the following best explains its cause**

1. a. Primary obstruction of the appendix
2. b. Sepsis
3. c. Peritonitis
4. d. Bowel rupture

**13. The diagnosis of appendicitis is largely based on which of the following**

1. a. Abdominal CT scan
2. b. Abdominal ultrasound
3. c. Signs and symptoms
4. d. Full blood count

**14. Which of the following should urgently be considered in a female of reproductive age pre- senting with acute pain in the right lower quadrant**

1. a. Splenic rupture
2. b. Ruptured ectopic pregnancy
3. c. Gastric rupture
4. d. Cholecystitis

**15. The Appendix is found in which part of the abdomen**

1. a. Lower left
2. b. Lower right
3. c. Upper right
4. d. Mid-abdomen

**16. Concerning signs and symptoms of acute appendicitis, which of the following constitutes some of the first signs**

1. a. Blood in stool
2. b. Abdominal pain/discomfort
3. c. Vaginal bleeding
4. d. Constipation

**17. Symptoms of appendicitis include**

1. a. Abdominal pain
2. b. Nausea
3. c. Anorexia
4. d. All the above

**18. Other conditions that could mimic pain of acute appendicitis include**

1. a. Inflammation of gall bladder
2. b. Stone in ureter
3. c. Inflammation of right colon
4. d. All the above

**19. Which of the following is not a sign of acute appendicitis**

1. a. Rovsing sign
2. b. Pointing sign
3. c. Murphys sign
4. d. Obturator sign

**20. A patient with inflammatory bowel syndrome was opened and an inflamed appendix found.**

**The treatment of choice is**

1. a. Appendectomy
2. b. ileocolic resection and anastomosis
3. c. Close the abdomen and start medical treatment
4. d. None of the above.
