# Supplementary figures and images for: Evaluating the Effectiveness of Interactive Virtual Patients for Medical Education in Zambia: Randomized Controlled Trial
Source: JMIR Med Educ. 2023 Jun 29;9:e43699. doi: 10.2196/43699 (PMC10501501; doi:10.2196/43699)

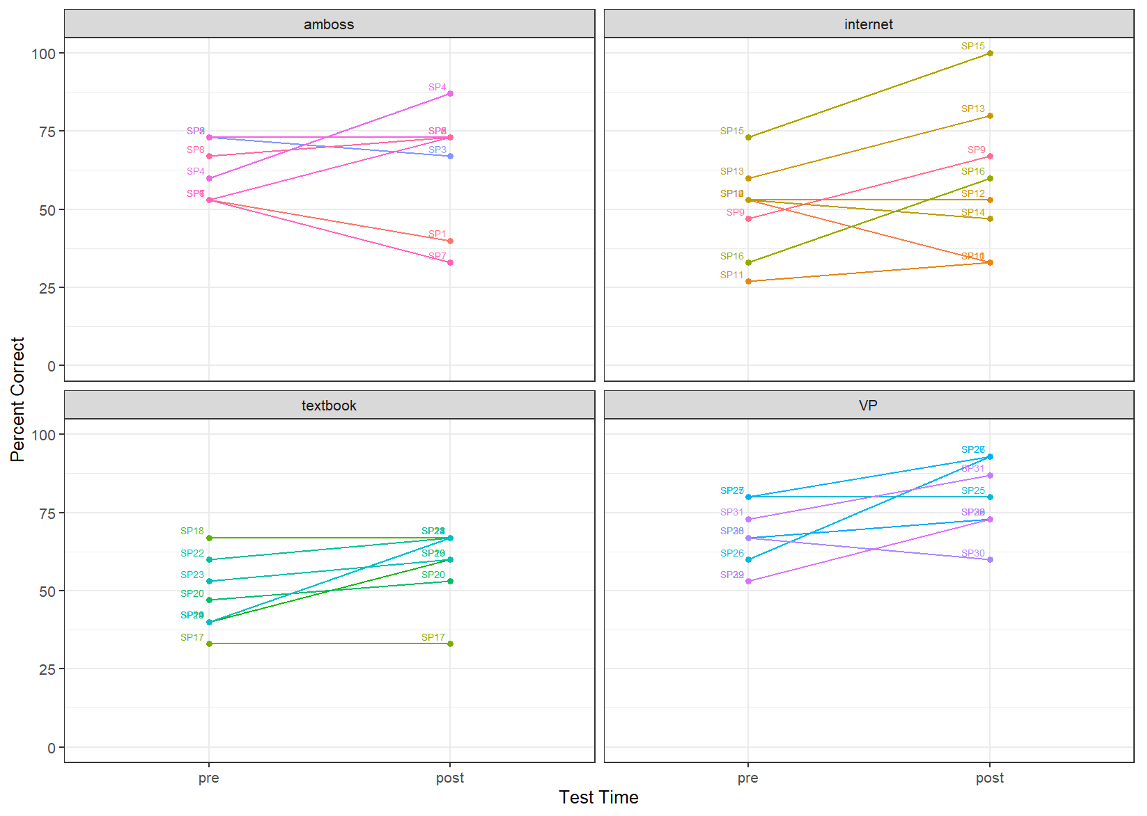

Supplement: Multimedia Appendix 6 [file mededu_v9i1e43699_app6.png]

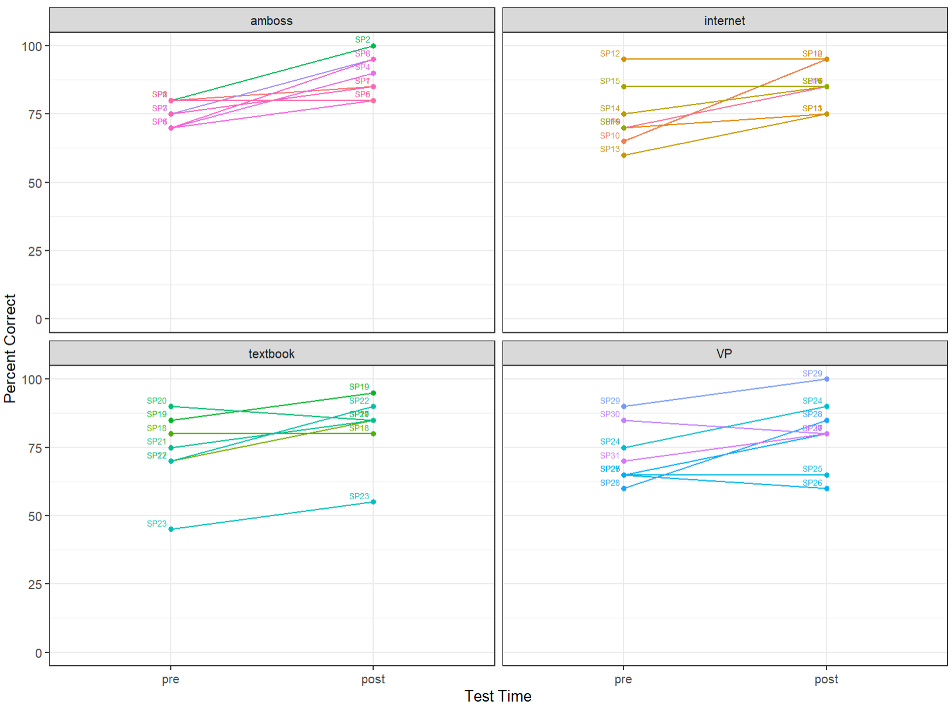

Supplement: Multimedia Appendix 7 [file mededu_v9i1e43699_app7.png]
